# Prosthetic Joint Infection Research Models in NZW Rabbits: Opportunities for Standardization—A Systematic Review

**DOI:** 10.3390/jfb15100307

**Published:** 2024-10-15

**Authors:** Julia L. van Agtmaal, Sanne W. G. van Hoogstraten, Jacobus J. C. Arts

**Affiliations:** 1 Laboratory for Experimental Orthopaedics, Department of Orthopaedic Surgery, Care and Public Health Research Institute (CAPHRI), Maastricht University Medical Centre, 6229 Maastricht, The Netherlands; julia.vanagtmaal@maastrichtuniversity.nl (J.L.v.A.); s.vanhoogstraten@maastrichtuniversity.nl (S.W.G.v.H.); 2 Department of Biomedical Engineering, Orthopaedic Biomechanics, Eindhoven University of Technology, 5612 Eindhoven, The Netherlands

**Keywords:** prosthetic joint infection, NZW rabbit, in vivo, antibacterial technologies, ARRIVE guidelines

## Abstract

Prosthetic joint infection (PJI) is a major complication following total arthroplasty. Rising antimicrobial resistance (AMR) to antibiotics will further increase therapeutic insufficiency. New antibacterial technologies are being developed to prevent PJI. In vivo models are still needed to bridge the translational gap to clinical implementation. Though rabbit models have been used most frequently, there is no consensus about methodology and measured outcomes. The PubMed, Scopus, and EMBASE databases were searched for literature on PJI in rabbit models. Data extraction included bias control, experimental design, and outcome measures of the NZW rabbit models in the articles. A total of 60 articles were included in this systematic literature review. The articles were divided into six groups based on the PJI intervention: no intervention used (21%), revision surgery (14%), prevention with only antibiotics (21%), prevention with surface modifications (7%), prevention with coatings (23%), and others (14%). Despite the current availability of guidelines and recommendations regarding experimental design, bias control, and outcome measures, many articles neglect to report on these matters. Ultimately, this analysis aims to assist researchers in determining suitable clinically relevant methodologies and outcome measures for in vivo PJI models using NZW rabbits to test new antimicrobial technologies.

## 1. Introduction

Up to 1 million total hip arthroplasties (THAs) and total knee arthroplasties (TKAs) are performed in the United States every year [1]. With an increasingly aging population, and rising risk factors such as malnutrition, obesity, or other co-morbidities such as osteoarthritis, the total number of THAs and TKAs is expected to grow even further [1,2,3]. Kurtz et al. [4] have stated that by 2030 this number is predicted to increase by 174% for THA and 673% for TKA. Though THA and TKA generally lead to patient satisfaction, prosthetic joint infection (PJI) following primary TKA and THA arises in 1–2% of all surgeries [1,5,6,7,8]. Delayed healing, inadequate functional outcome, decreased quality of life, and increased mortality occur as a consequence of PJI [6,9]. PJI increases hospital resources immensely, increasing the economic burden on the healthcare system [10]. PJI is a significant contributor to primary THA and TKA failure and is responsible for 30–40% of all failures in revision THAs and TKAs [1,5,7]. With an increasing number of THAs and TKAs, the incidence of PJI will likely also further increase.

Currently, most PJIs are caused by the Gram-positive bacteria *Staphylococcus aureus* (*S. aureus*) and *Staphylococcus epidermidis* (*S. epidermidis*) [7,9,11]. However, the rate of PJIs caused by Gram-negative bacteria has increased, reaching up to 40% in TKA and total shoulder arthroplasty (TSA) [12].

Currently, the standard of care to treat PJI and biofilms is to start with high-dose antibiotics [13]. If the infection is not cleared by antibiotics alone, the surgical options are debridement with a one- or two-staged revision, or debridement, antibiotics, and implant retention (DAIR) procedures [13,14]. However, bacteria can form biofilms: an accumulation of tightly packed bacteria on the implant, encased by an extracellular matrix that protects the bacteria [15]. Once bacteria have adhered to the surface of the implant, and have won the so-called race for the surface and colonized the implant surface, biofilm formation happens rapidly and hampers host tissue cell function [16].

Biofilm formation is not limited to the implant; it can be dispersed to the bone, bone cement, synovial fluid, and tissue surrounding the bone [7]. This extracellular matrix mainly consists of proteins, polysaccharides, and extracellular DNA [17]. Biofilm formation consists of four stages: bacterial adhesion, biofilm formation, biofilm maturation, and biofilm dispersal. Biofilm dispersal means that not only does the PJI persist, but other body sites can also become infected [15]. The biofilm forms a physical barrier, slowing antibiotic diffusion and hindering the patient’s internal immune cells, allowing the PJI to continue [13,15,18,19]. Bacteria within a biofilm are 500–5000 times less susceptible to antibiotics as compared to planktonic bacteria, creating “persister cells” that are dormant, highly tolerant to antibiotics, and reactivated when treatment has stopped [13]. Moreover, due to systemic overuse and misuse of antibiotics, the incidence of antimicrobial resistance (AMR) to antibiotics is growing fast, putting patients with PJIs at risk of therapeutic insufficiency [9,20,21]. PJI and AMR are, therefore, an ever-growing threat, and new technologies are needed to prevent bacterial attachment and biofilm formation on the implant surface.

Research into developing treatment or prevention options for PJI and biofilm formation has become increasingly prevalent, focusing on new antibacterial coatings, surface designs, or other compounds to protect implants, with or without antibiotics. There are three main antimicrobial mechanisms for antibacterial coatings: anti-fouling or non-adhesive, contact-killing, and antimicrobial-releasing [22]. Multiple in vitro test methods are available to test these technologies, where the choice of in vitro test depends on the antimicrobial mechanism [22]. However, studies have shown that in vitro results do not necessarily translate to in vivo results [23,24]. This is due to a lack of standardization in in vitro testing, a lack of clinically relevant test protocols, and the complexity of in vivo systems [23]. Several important in vivo factors cannot be replicated or integrated easily in vitro. First, in vitro tests are often performed under static conditions, whereas fluid flow may influence the in vivo results [25]. Also, the immune response is excluded in in vitro testing, neglecting the effect of the antimicrobial on the immune system and the impact of the immune system on biofilm formation [26]. Furthermore, antimicrobials may bind to blood serum proteins, which can alter their effect [27]. In addition, quorum sensing is a communication system used by bacteria to control biofilm formation, which is often disregarded in vitro [25]. Last, a high hammering force is applied during THA and TKA surgeries, possibly creating microfractures and affecting the implant coating by scratching or damaging them [28]. Testing the mechanical properties of antibacterial coatings is often overlooked. Therefore, preclinical in vivo models are needed to study both the host response and integration of the implant, as well as the interaction with pathogens, to bridge the translational gap to the clinic [24]. Additionally, the Food and Drug Administration (FDA) and the Medical Device Regulation (MDR) emphasize the importance of in vivo experimentation as a critical step before clinical studies. In vivo tests are essential to test the biocompatibility, safety, infection prevention efficacy, and tissue response to the implant and the new antimicrobial compounds.

Rabbits have been used in up to 35% of musculoskeletal in vivo models, and most frequently to study PJI in vivo [29,30,31]. In particular, New Zealand White (NZW) rabbits are most commonly used [32]. They are docile, non-aggressive, and easy to handle and observe, yet still large enough to implement orthopedic implants [30,33]. The bone and joint biology and the response to infections of rabbits mimic that of human joint infections. This similarity in infection susceptibility, pathogenesis, and immune response is crucial when evaluating PJI and the effectiveness of antimicrobial treatments for clinical applications [30,34]. In contrast, rats and mice, the most frequently used animals in testing, are less susceptible to infection than rabbits [32,34]. Also, rabbits are relatively low in cost and take up limited space, compared to large animal models [33].

Though rabbit models have been used widely to study PJI and new antimicrobial technologies, there is no consensus about the exact methodology and outcome measures [24,35]. Moriarty et al. [24] state that many common errors are still made in these in vivo studies, such as not quantifying the bacteria. Due to these discrepancies in methodologies and outcomes, antibacterial techniques cannot be compared to one other, and it remains unclear when a technique’s antibacterial efficacy is good enough to progress to a larger animal model or clinical studies. Furthermore, papers often fail to document all aspects of their studies according to the ARRIVE guidelines, a checklist of recommendations for the full and transparent reporting of research involving animals [36]. Due to this unclarity, more rabbits are currently being used than necessary. Reporting information is important to avoid the repetition of experiments and unnecessarily using animals in inconclusive research [37]. It is important to use animals responsibly, improve the treatment of the animals, and increase the quality of the studies by implementing Russel and Burch’s 3R principles: reduction, refinement, and replacement [38].

A review of NZW rabbit models to study PJI and treatment interventions, and the applied methodologies of these studies, is necessary to increase standardization in these models. Therefore, this systematic review will focus on the methodology and measured outcomes in in vivo models that mimic PJI in NZW rabbits. First, similarities in methodologies and outcome parameters among the included studies will be identified. Second, areas that present opportunities for methodological standardization will be defined and discussed. The bias control, experimental design, and experimental outcomes of all included studies will be assessed to do this. Ultimately, this analysis aims to assist researchers in determining suitable clinically relevant methodologies and outcome assessments in an in vivo PJI model using NZW rabbits to test new antimicrobial technologies. This can result in more standardization and a better assessment of the antibacterial potential of new technologies to prevent or treat PJI using in vivo NZW rabbit models. To conclude, this systematic review will provide a comprehensive analysis of current methodologies and outcomes measured in NZW rabbit PJI models, aiming to enhance standardization and improve the evaluation of antimicrobial interventions.

## 2. Methods

This literature search was conducted according to the Preferred Reporting Items for Systematic Reviews and Meta-Analysis (PRISMA) guidelines [39], and it was registered in the International Prospective Register of Systematic Reviews (PROSPERO, ID number CRD42024411818, York, UK). The PubMed, Scopus, and EMBASE databases were used to search the literature on 8 May 2024. The English language was used as a selection filter for the database searches. For PubMed, the following search string was used: ((“prosthesis-related infections” [MeSH]) OR “PJI” OR “prosthetic joint infection” OR “joint replacement infection” OR “arthroplasty infection” OR “implant infection” OR ((“osteomyelitis” [MeSH]) AND “implant”) OR (“orthopaedic infection” AND “implant”) OR (“bone infection” AND “implant”) OR “prosthetic infection” OR “peri-prosthetic infection” OR “implant-related infection” OR “DAIR”) AND ((“rabbits” [MeSH]) OR (“lagomorpha” [MeSH])) AND (“experimental model” OR (“models, animal” [MeSH]) OR “preclinical model” OR “in vivo”). This search string was adapted to conform to the Scopus database and can be found in Appendix A. Lastly, the search for EMBASE is shown in Appendix A, Table A1. Osteomyelitis and DAIR were included in the search strings, as certain studies utilize models pertinent to PJI, even though they are not the focus of this research.

Using Covidence™ (Veritas Health Innovation, Melbourne, Australia) [40], an online software platform designed to streamline the process of conducting systematic reviews, all article abstracts were screened individually and blinded by two reviewers (J.v.A. and S.v.H.). Articles focusing on rabbit models used to study orthopedic implant infections were included for further full-text screening. During the full-text screening, articles were excluded based on multiple criteria. Firstly, the applied exclusion criteria based on publication criteria were used: articles published before 2000, review articles or discussions, conference abstracts, letters to the editor, studies that were not published in peer-reviewed journals, non-English studies, and non-retrievable studies. Secondly, based on study type, the following exclusion criteria were applied: in vitro studies, in vivo studies on animals other than NZW rabbits, and clinical trials. Thirdly, based on the relevance of the articles, the applied exclusion criteria were infections unrelated to bone or orthopedics, studies that investigate interventions that are not relevant to PJI, studies that use implants that are not for joints, studies about FRI or DAIR that did not use models relevant to PJI, studies that do not use a bacterial inoculum or that grow biofilm in vitro before the operation, and studies that do not use an implant. Lastly, based on reporting criteria, the applied exclusion criteria were studies that do not report on outcomes such as infection rates, microbiological findings, histopathological findings, and implant-related complications. Articles that investigated bone cement or bone void-filling biomaterials were only included if their intended use was a two-stage revision as a solution for PJI. If the two independent researchers could not decide on conflicting articles, a third independent reviewer (J.A.) evaluated the article. Finally, after the screening process, data extraction was performed, including bias control, experimental design, and outcome measures. Data extraction was performed by one reviewer, and 33% of the articles were cross-checked by the second reviewer.

## 3. PRISMA Results and Data Extraction

**Figure 1:**
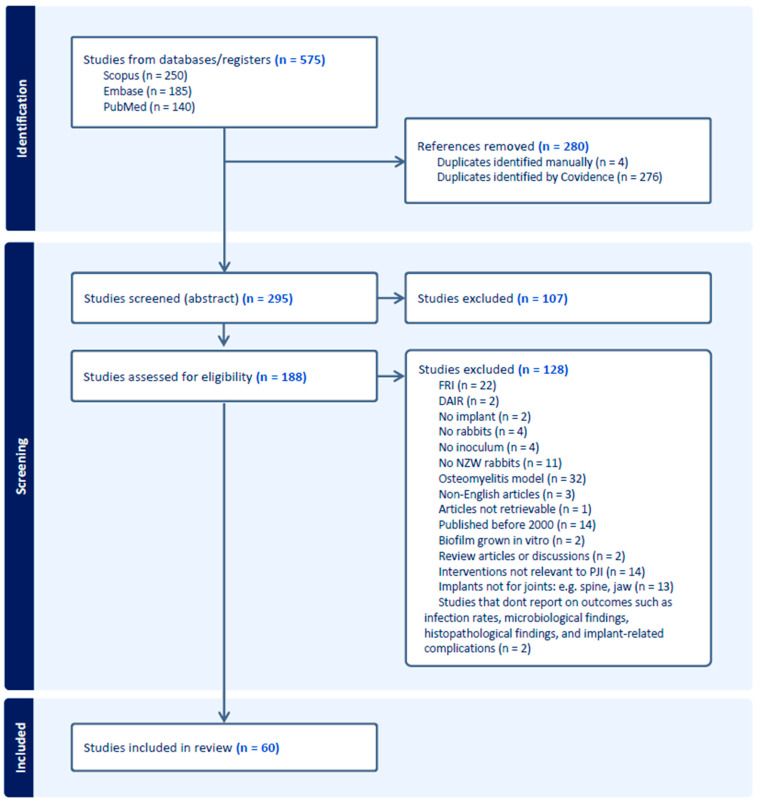
PRISMA flow diagram of article selection. The database search resulted in a total of 575 studies. After duplicate removal and screening, 60 studies were left for data extraction.

A total of 575 studies were found on the three databases PubMed, Scopus, and Embase, of which 295 remained after duplicate removal (Figure 1). During the abstract screening, 107 studies were excluded due to their content being unrelated to rabbit models used to study orthopedic implants and infections. Of these 107 studies, a consensus was reached for all but four by the first two researchers. Assessment of these four articles was completed by the third reviewer (J.A.). Of the remaining 188 studies of which the full texts were assessed for eligibility, 128 were excluded (Figure 1). Only four of these studies had to be assessed by the third reviewer (J.A.) due to a lack of consensus among the first two researchers. The main reasons for exclusion were that the articles studied osteomyelitis (*n* = 32) or FRI models (*n* = 22). This left 60 studies for extraction. The first researcher (J.v.A.) carried out the extraction of the 60 articles. The second researcher (S.v.H.) cross-checked 20 of these articles. The data extracted by both researchers was highly comparable; therefore, not all articles were extracted by the second researcher.

**Figure 2:**
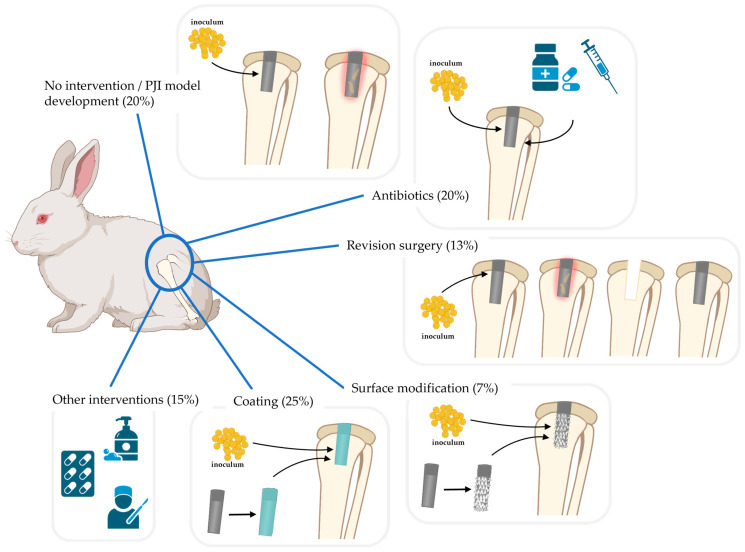
Interventions used in the NZW rabbit PJI models in the articles included in this review. Articles studied no intervention/PJI model development (20%); antibiotics alone as prevention for PJI (20%); revision surgery as treatment for PJI (13%); surface modification of the implant as prevention for PJI (7%); coating on the implant as prevention for PJI (25%); or other interventions (15%). Created with Biorender.com (accessed on 14 May 2024).

The data extraction was divided into three parts: bias control, experimental design methodology, and measured outcomes. As is displayed in Table 1, the section on bias control focused on the blinding and randomization of the studies, the rabbit characteristics, humane endpoints, and care of the rabbits. The section on experimental design methodology is described in Table 2 and Table 3. This section includes the aim and duration of the study, and information about the inoculum, implant, interventions, and experimental groups, including dropout percentage per group. The measured outcomes of each study are shown in Table 4 and Table 5. This table focuses on bacterial culture, health monitoring, hematology, histology, and staining. Based on the interventions against PJI that were found in all included articles, the articles were divided into six groups: no intervention used against PJI, revision surgery, prevention of PJI with only antibiotics, prevention of PJI with surface modifications, prevention of PJI with coatings, and others (Figure 2).

## 4. NZW Rabbit Models to Investigate PJI

This systematic review focused on in vivo models to investigate PJI and antimicrobial technologies in New Zealand White (NZW) rabbits. The 60 included studies were grouped based on the intervention used against PJI. This categorization showed that 20% of the articles used no intervention against PJI in their research (Figure 2), which implies that all these studies were developing or validating a new rabbit PJI model. This number of model development papers emphasizes the need for standardized guidelines to set up a suitable, clinically relevant, in vivo PJI model in NZW rabbits. This need for standardized criteria was previously highlighted during the 2023 international consensus meeting on musculoskeletal infection (MSKI) [41]. Standardization will result in a reduction in animal use. Furthermore, with the rise of antimicrobial resistance (AMR) to antibiotics [9,20,21], it is noteworthy that 20% of all articles investigated existing and on-the-market antibiotics as the only antimicrobial agent. The other studies were categorized as revision surgery (13%), which included one- and two-stage revision; surface modifications of the implant (7%), like polishing; implant coatings (25%); and other interventions that did not fit the different categories (15%). All studies were reviewed on their bias control, experimental design, and reported outcomes.

### 4.1. Bias Control

Bias should always be avoided in research [24]. Table 1 shows how bias control was handled in all reviewed studies. Bias control in in vivo studies is essential for maintaining research integrity, validity, and ethical conduct. Furthermore, bias control enhances the reliability and reproducibility of research findings. Therefore, translating results from preclinical in vivo animal studies towards clinical studies depends on bias control [23,42]. First, as stated by Moriarty et al. [24], blinding and randomization are the minimal requirements to limit bias. Second, as elucidated by both the ARRIVE guidelines [43,44] and Moriarty et al. [24], reporting the animal characteristics in in vivo experiments is highly recommended to limit the effect of potential bias. Animal characteristics include the species, strain, sex, age or skeletal maturity, and weight of the animal [24,43]. These guidelines also highlight the importance of including the housing and husbandry details of an in vivo experiment [24,43]. Husbandry details also contain welfare-related assessments, including humane endpoints at which point the suffering of the rabbits is no longer justified by the scientific value the experiment provides [43,45].

#### 4.1.1. Blinding and Randomization

**Figure 3:**
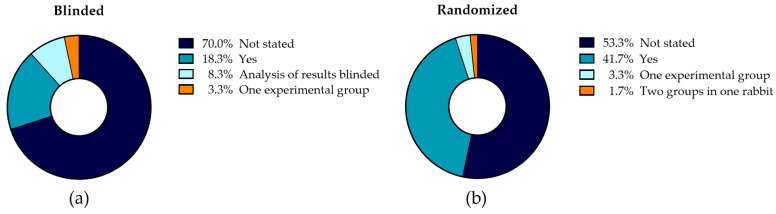
Analysis of bias control Table 1, including: (**a**) blinding of the studies; (**b**) randomization of the studies.

The risk of bias is highly dependent on blinding and randomization in studies. As stated by Bespalov et al. [46], blinding and randomization are necessary if the study results have an impact on decision-making and cannot be easily repeated due to ethical or resource-related reasons. Both requirements are present in this review, as rabbit in vivo studies are essential before proceeding to clinical studies, and the number of used animals in research should be reduced as much as possible [46,47]. Two types of blinding should be performed: blinding the researchers performing the surgery, which minimizes the chance of performance bias, and blinding the researchers performing the analysis of the results, minimizing the risk of detection bias [46,48]. Figure 3a illustrates that in 70% of the included studies, it was not stated if the involved researcher performing the surgery was blinded to which implant, treatment, and inoculum they were inserting in the rabbits, or if the results were analyzed blinded. Only 18.3% of the studies stated that the experiments were performed blinded, and in 8.3% of the studies only the results were blinded to the researchers analyzing the data. In 3.3% of the studies, there was only one experimental group, making blinding unnecessary. This lack of blinding of the studies, or the reporting thereof, allows room for conscious or unconscious biases to influence results. Since researchers frequently face a conflict of interest in their eagerness to get their products to market, it is essential to avoid any biases.

Using randomization to create experimental groups allows researchers to use probability theory to determine if outcome differences are due to chance [46]. Furthermore, randomization minimizes the chance of selection bias, reducing the chance that rabbits with preferable features are grouped. As can be seen in Figure 3b, 53.3% of the studies did not report on the randomization of their experiment. A form of randomization was used in 41.7% of the studies to assign experimental groups to the rabbits. The remaining 5% consisted of studies in which randomization was no option because there was only one experimental group, or two experimental groups were implemented into one rabbit. The lack of randomization in more than half of the studies diminishes comparability between experimental groups. As stated before, the scarcity of documentation, blinding, and randomization found in the reviewed studies weakens the scientific integrity, reliability, and reproducibility of the studies. Notably, Laajala et al. [42] state that preclinical studies also benefit from implementing the best practices of human clinical trials. These best practices include blinding and randomization, increasing translation from preclinical to clinical studies. Variability and false positives in the intervention effects are reduced when creating randomized, blinded groups that are representative of the population that are handled and treated similarly.

#### 4.1.2. Rabbit Characteristics

**Figure 4:**
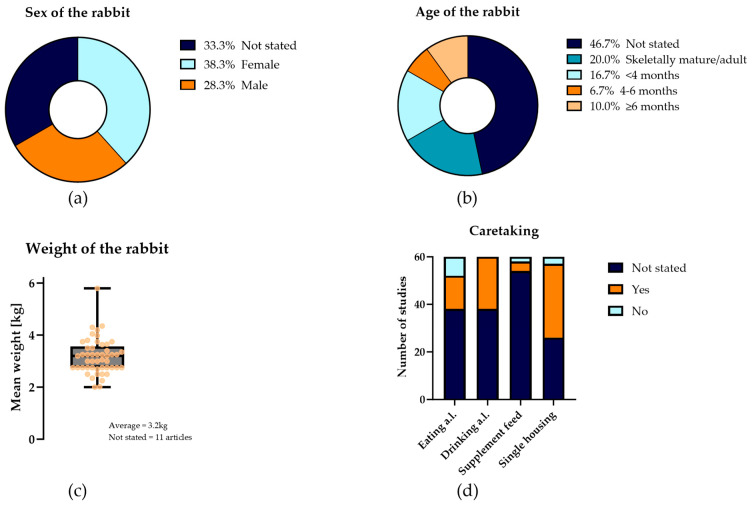
Analysis of bias control Table 1, including (**a**) the sex of the rabbits included in the studies; (**b**) the age of the rabbits included in the studies; (**c**) the weight of the rabbits included in the studies; (**d**) caretaking of the rabbits included in this study, including if they could eat and drink ad libitum (a.l.), if they were provided with supplemental feeding when necessary, and if they were single housed or not.

Common sources of variation within preclinical models are genetic differences, sex, age range, and weight [42,49]. These can be found in Table 1 (‘rabbit characteristics’). As all included studies in this systematic review use NZW rabbits, genetic differences are limited as much as possible. As elucidated in Figure 4a–c, the sex, mean weight, and age of the rabbits are not stated in 33.3%, 18.3%, and 46.7% of the studies, respectively. The ARRIVE guidelines highly recommend including these four characteristics in animal research [43,44]. Variation in study results within experimental groups may be limited by defining which rabbits to include in the experiment and creating balanced experimental groups [42]. Unfortunately, as this information is unknown in the studies where the characteristics are not stated, this creates uncertainty about whether disparities between experimental groups are due to the different treatments or due to differences in animal characteristics.

The sex of the animals is a biological variable in research outcomes, and, as stated by the National Institutes of Health (NIH) and ARRIVE guidelines, should be taken into account and reported in all in vivo and clinical studies [43,44,50]. Males and females may differ in physiology, metabolism, hormonal profiles, and cellular functions, which can impact experimental outcomes [50]. Kunutsor et al. [51] emphasize this in their study, where they determined that males have a higher chance of developing PJI compared to females. Studying only one sex can increase bias, limit the generalizability of the results, and decrease reproducibility [50,52]. Furthermore, excluding males or females may lead to potential harm or suboptimal outcomes for the excluded population and, as follows, impair the translation to the clinic [53]. Multiple studies therefore advise including both sexes in the design of preclinical studies, to account for differences between the sexes [43,44,50,53,54].

However, no study analyzed in this systematic review included both sexes. As shown in Figure 1, most studies (38.3%) used only female rabbits, 28.3% used only male rabbits, and 33.3% did not state the sex of the rabbits. Though using both sexes in animal models is advised, several arguments exist for using only one sex. First, rabbits that are housed in pairs need to establish a hierarchy. Thurston et al. [55] demonstrated that when housed in pairs, 1% of the female pairs and 20% of the male pairs had to be separated due to fighting. Second, although males have a higher chance of developing PJI as compared to females, Mironenko et al. [56] concluded that the incidence of treatment success does not differ between the sexes in humans. Third, the higher percentage of studies that use female rabbits might be explained by their larger size, making handling the rabbits easier [57]. However, no major anatomical differences exist between surgical areas between the sexes [57,58]. Fourth, in some studies, male animals are preferred due to their hormonal stability; however, female rabbits are induced ovulaters, meaning they remain in estrus until copulation, after which ovulation starts [59]. Therefore, their hormonal balance is relatively stable as well. Last, as stated previously, there may be differences in response to PJI treatments between males and females due to, e.g., their hormonal profiles and immune response [50]. Due to this possible difference in response between male and female rabbits, the number of rabbits per experimental group should also increase, significantly raising the study’s costs. Ultimately, researchers should specify which sex they utilize in their research and provide a rationale for their choice, conforming with the ARRIVE guidelines.

The age and starting weight of the rabbits are important factors in defining the skeletal maturity of the rabbits. Skeletal maturity is important, as a mature bone structure is essential for proper implant placement and fixation. Skeletal maturity is reached at five to six months of age [35]. Furthermore, young rabbits are less prone to infections, as maternal antibodies still protect them [60,61]. Marchandeau et al. [60] state that these antibodies both prevent infections and allow attenuated infections that activate the immune system of the young rabbit. Differentiating between the effect of these maternal antibodies and the antimicrobial compound that is tested is therefore challenging. Also, as stated by Moriarty et al. [23], PJI is most prevalent in the older human population, resulting in possible co-morbidities and altered immune systems. Using young rabbits complicates clinical translation, as they do not represent the target patient group. Masoud et al. [62] measured the growth, weight, and tibial length over 34 weeks. They concluded that skeletal growth was complete at 28 weeks. At sixteen weeks, the mean body weight was 72%, the mean body length at 91%, and the mean tibial length at 94% of the adult value. Figure 4b shows that 16.7% of the studies used rabbits younger than four months, meaning they had not yet skeletally matured. A total of 35% of the studies used skeletally mature rabbits: they were either four to six months (6.7%), older than six months (10%), or it was stated that they were skeletally mature (20.0%). However, it would be better for papers claiming skeletal maturity to specify the exact age and starting weight of the rabbits for standardization purposes. To confirm the skeletal maturity of the rabbit, it would be most optimal to perform an X-ray to determine if the growth plates are closed [63]. The weight of adult NZW rabbits may range from 2 to 6 kg [63]. No studies included in this review reported the starting weight to be below this; however, 18% did not report on the rabbits’ starting weights at all. As with the other rabbit characteristics, reporting of the age and weight of the rabbits is inadequate, despite their potential influence on the results of the experiments.

#### 4.1.3. Housing and Husbandry

As stated previously, housing and husbandry details are important to include in performing and reporting in vivo experiments, both for the scientific value and the validity, as well as from an ethical perspective [24,43]. Housing and husbandry include the humane endpoints and the caretaking of the rabbits, as found in Table 1. For scientific value and validity, it is important to set the endpoints, as letting rabbits be included in results that are suffering too much or have comorbidities might influence the results [43,45]. Furthermore, the caretaking protocol for the rabbits should be set beforehand and precisely documented. This might otherwise result in preferential treatment of the experimental group receiving the antibacterial technique. Voehringer et al. [56] state that study designs can be more efficient. Research methods can be improved by including sufficient animal care and husbandry, which is linked to reduction and refinement [38]. A gold standard publication checklist, published by Hooijmans et al. [64,65], which integrates the reduction and refinement principle of the 3Rs, also emphasizes the need for animal husbandry and care standardization, including housing, nutrition, and water intake. Figure 4d shows that the studies included in this review often fail to report on caretaking details. It was examined whether studies reported the availability of water and food ad libitum (a.l.), the housing conditions of the rabbits (single or group housing), and whether supplemental feeding was provided when rabbits experienced significant weight loss. The details were not stated in 61% of the studies for water and food a.l., 40% for single housing conditions, and 89% for supplemental feeding.

As stated by the European Parliament in their directive on animal protection [66], death as an endpoint to a procedure should be avoided as far as possible and replaced by earlier, humane endpoints. The severity and duration of pain, distress, and suffering of the animals due to adverse effects of the surgery or treatment should be minimized and should justify the scientific value added by the research, in line with the 3Rs [67,68]. A total of 75% of the included studies in this research did not report on their humane endpoints. Several humane endpoints have been published online for when this pain and suffering no longer justify the scientific value added by the research [68]. Several humane endpoints often found in infection research in rabbits, as shown in this review, are bone fracture, severe weight loss, and infection outside the joint. Ultimately, reporting of the humane endpoints and caretaking is essential to minimize bias.

**Table 1:** Bias control extraction results. Abbreviations used: ns = not stated.

Reference	Bias Control		Rabbit Characteristics			Humane Endpoints	Caretaking			
	Blinded	Randomized	Sex (m/f/ns)	Age Range or Skeletally Mature (s.m.)	Weight Range (kg))	1. Fracture 2. Weight Loss (%) 3. Infection Outside Joint 4. Persistent Swelling and Discharge 5. Other Signs of Systemic Infection (Fever, Depression)	Eating a.l.	Drinking a.l.	Supplemental Feed	Single Housing
**No intervention used**										
[69]	Yes	ns	m	ns	Mean 4.2 kg	Dehiscence of surgical wound, screw exposure, MRSA expression	Yes	Yes	ns	Yes
[70]	Histology grading	ns	f	s.m.	Ns	2 (>10% in 2 weeks), 3, 4, 5	ns	ns	ns	Yes
[71]	Only 1 group	Only 1 group	f	8–12 weeks	2–3.5 kg	ns	Yes	Yes	ns	Yes
[29]	Yes	Yes	f	6 months	3.5–4 kg	1, 2 (>20%), 3	Yes	Yes	Yes	No
[72]	ns	Yes	f	ns	3.5–4 kg	1, 2 (>20%), 3	Yes	Yes	Yes	No
[73]	ns	ns	m	4 months	3–3.6 kg	ns	No	Yes	ns	Yes
[74]	ns	ns	f	74–120 days	2.9–3.5 kg	ns	ns	ns	ns	Yes
[75]	ns	Yes	f	74–120 days	1.7–3.0 kg	ns	Yes	Yes	ns	Yes
[76]	Data analysis	Yes	m	ns	3.0–3.5 kg	ns	ns	ns	ns	Yes
[77]	ns	Yes	f	~180 days	2.5–3.0 kg	1, not developing an infection in the infection group	No	Yes	ns	Yes
[78]	PET/CT results	ns	ns	ns	ns	ns	ns	ns	ns	Yes
[30]	X-ray evaluation	ns	f	ns	ns	2 (>20%)	Yes	Yes	Yes	Yes
**Revision**										
[79]	ns	Yes	ns	6–8 months	5.8 ± 0.24 kg	2 (>20%), 4, complete loss of function of the left limb, rejection of nutriment	ns	ns	ns	Yes
[80]	ns	ns	ns	ns	ns	ns	Yes	Yes	No	Yes
[81]	ns	Yes	f	ns	2.5–3 kg	ns	ns	ns	ns	Yes
[82]	ns	Yes	ns	ns	ns	ns	ns	ns	ns	ns
[83]	ns	ns	f	ns	~3 kg	No PJI 1 week after inoculation	Yes	Yes	No	ns
[84]	ns	ns	f	Adult	3000–3500 g	ns	No	Yes	ns	Yes
[85]	Yes	Yes	f	ns	3–4 kg	ns	ns	ns	ns	ns
[86]	ns	ns	f	Adult	2840–3100 g	ns	ns	ns	ns	ns
**Prevention: antibiotics only**										
[87]	ns	ns	m	Adult	3–4.5 kg	3	ns	ns	ns	ns
[88]	ns	Yes	f	ns	1.22–3.02 kg	ns	ns	ns	ns	ns
[89]	ns	Yes	ns	ns	3–4 kg	ns	ns	ns	ns	ns
[90]	ns	Yes	ns	ns	2.5–3 kg	ns	ns	ns	ns	Yes
[91]	ns	ns	m	Adult	2.7 ± 0.2 kg	ns	ns	Yes	ns	Yes
[92]	Yes	ns	f	7 months	3.0–3.5 kg	3	ns	ns	ns	ns
[93]	ns	Yes	m	ns	1.8–2.2 kg	ns	ns	ns	ns	ns
[94]	Yes	ns	ns	ns	2.2–2.8 kg	ns	ns	ns	ns	Yes
[95]	ns	ns	ns	ns	2.5–3 kg	ns	ns	ns	ns	ns
[96]	ns	ns	ns	ns	ns	ns	ns	ns	ns	ns
[97]	ns	Yes	ns	ns	2.5–3 kg	ns	ns	ns	ns	Yes
[98]	ns	Yes	f	>6 months	3.0–3.5 kg	ns	ns	ns	ns	ns
**Prevention: surface modification**										
[99]	ns	ns	m	ns	ns	ns	ns	ns	Yes	Yes
[100]	ns	ns	f	34 weeks	3.98 ± 0.54 kg	1, 2 (% ns), 3	ns	ns	ns	Yes
[101]	ns	ns	m	26 ± 8 weeks	3.7–3.9 kg	2 (% ns), profoundly decreased general condition	ns	ns	ns	Yes
[102]	ns	Yes	m	s.m.	3.2 ± 0.2 kg	ns	Yes	Yes	ns	Yes
**Prevention: coating**										
[103]	Only 1 group	Only 1 group	ns	ns	3500–5200 g	Wound dehiscence with implant exposure	ns	ns	ns	Yes
[104]	Yes	ns	f	16 weeks	ns	2 (>15%), 3, Shock	No	Yes	ns	No
[105]	Yes	ns	ns	ns	3.7–4.4 kg	ns	ns	ns	ns	ns
[106]	Histology grading	Yes	ns	Adult	3000–3500 g	ns	Yes	Yes	ns	Yes
[107]	ns	Yes	m	Adult	3000–3500 g	ns	No	Yes	ns	Yes
[108]	ns	ns	f	s.m.	4.3 ± 0.4 kg	1, 2 (>10% in 2 weeks), 3, 4, local infection with severe lameness	ns	ns	ns	ns
[109]	Yes	ns	f	ns	2.6–3.5 kg	ns	No	ns	ns	Yes
[110]	ns	ns	f	ns	ns	ns	ns	ns	ns	ns
[111]	Yes	ns	f	s.m. *^1^	2900–3600 g	ns	No	Yes	ns	Yes
[112]	ns	ns	m	2 months	~3 kg	ns	Yes	Yes	ns	ns
[113]	ns	Yes	m	8 months	2.5–3 kg	ns	ns	ns	ns	ns
[114]	ns	Yes	m	3 months	~2.5 kg	ns	ns	ns	ns	ns
[115]	ns	Yes	m	ns	2.5–3.0 kg	ns	ns	ns	ns	ns
[116]	ns	ns	m	3 months	2–3 kg	ns	Yes	Yes	ns	Yes
[117]	ns	ns	ns	3–4 months	2.5 ± 0.5 kg	ns	ns	ns	ns	ns
**Other**										
[118]	Yes	Yes *^2^	ns	ns	3.2–4.1 kg	ns	Yes	Yes	ns	ns
[119]	ns	Yes	ns	ns	ns	ns	ns	ns	ns	ns
[120]	Yes	ns	m	s.m.	3.0–3.5 kg	ns	No	Yes	ns	Yes
[121]	ns	ns	m	Adult	3045–4225 g	ns	ns	ns	ns	ns
[122]	ns	ns	ns	8–12 weeks	ns	ns	ns	ns	ns	ns
[123]	ns	Yes	ns	4–5 months	2.0–2.5 kg	ns	ns	ns	ns	ns
[124]	ns	Yes	ns	ns	2.5–3.5 kg	ns	Yes	Yes	ns	Yes
[125]	ns	ns	f	70–100 days	2.5–3.0 kg	ns	ns	ns	ns	Yes
[126]	ns	Yes	ns	ns	2.5–3.0 kg	ns	ns	ns	ns	ns

*^1^—The article says ‘young adult’; however, the authors have been contacted and have confirmed that the growth plates were closed. *^2^—Each rabbit has a randomly assigned control and experimental knee.

### 4.2. Experimental Design

Translating the results from in vivo experiments on antibacterial technologies to the clinic is challenging [23,24]. Currently, in vivo preclinical results do not consistently anticipate clinical outcomes [23]. Choosing the right experimental design and methodology is crucial in bridging this gap as much as feasible. In addition, the research aim is inextricably connected to the experimental design. A first proof-of-concept in vivo experiment might involve different parameters compared to a final preclinical study before human trials. Table 2 and Table 3 illustrate the experimental design parameters of the studies analyzed in this review, including the study’s duration, inoculum and implant details, and the interventions against PJI tested in the research and in which experimental groups they were tested.

#### 4.2.1. Study Duration

**Figure 5:**
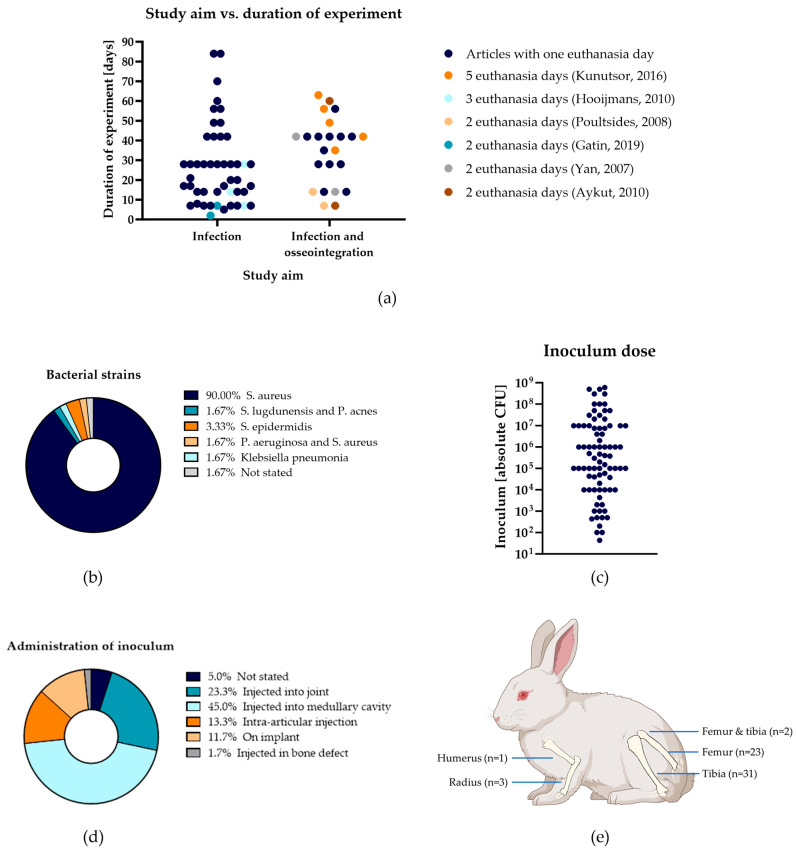
Analysis of the experimental design of the articles included in this review, including: (**a**) the duration of the experiments from inoculation to euthanasia for studies that only investigated infection (**left**) and studies that investigated infection and osseointegration (**right**), and articles that studied multiple study durations were colored separately; (**b**) bacterial strains used in the studies; (**c**) the inoculum dose [absolute CFU] that was used to inoculate the rabbits; (**d**) the bone in which the implant was inserted; and (**e**) the way in which the inoculum was administered to the rabbit ([52,65,74,82,87,89]). Created with Biorender.com (accessed on 20 August 2024).

As stated above, the study aim is the leading factor determining the experimental design. This especially applies to the duration of the study. The duration of the study is dependent on the mode of action of the antibacterial technology and on what outcome measures are evaluated. The durations of the experiments in the included studies in this research are illustrated in Figure 5a, and they range from 84 days [79,103] to 2 days [110]. Studies that only look at infection progression, and not osseointegration, most often last 27 days (*n* = 11), 7 days (*n* = 8), or 14 days (*n* = 6). For a first pilot in vivo study for surface modifications, or contact-killing or anti-fouling coating, where the antibacterial substance should be immediately active, and that only investigates the antibacterial activity of the coating, 7 to 14 days might be sufficient. For antibacterial-releasing coatings or revision surgeries, the duration of the experiment is dependent on the time to establish an infection and the activation or release of the antibacterial substance. If the short-term functioning of the antibacterial substance has been established, testing the long-term effect should also be evaluated.

An ideal duration should be found for the antibacterial compound to work, to initiate an infection in the control group, and not prolong unnecessary animal suffering. Remarkably, two studies lasted 84 days. One of these studies was by Brunotte et al. [79], where the study was divided into four parts of 4 weeks each: initial insertion of the implant and inoculum; revision stage 1 with implant removal, debridement, and insertion of a spacer; revision stage two with spacer removal and insertion of a new implant; and euthanasia. Bitika et al. [103] also chose 84 days, as they state that this time is needed for mature osseointegration of titanium implants. However, in their study, the only outcome measures are bacterial culture and health monitoring, with no quantification of osseointegration. For the study of Brunotte et al. [79], a duration of 84 days might be justifiable, as this is needed to test the intended use of their antibacterial compound. However, for Bitika et al. [103], their antibacterial compound and study outcomes do not justify this prolonged animal suffering. In contrast, Neut et al. [110], had a study duration of only two and seven days, although they expected their CFU count for the experimental groups to be <1 log for both time points. The results from day two and day seven were comparable, and the need for the two time points is not elucidated. At two days post-surgery, the rabbits might still be recovering, which could affect some results, such as certain hematology values, making them unreliable.

In addition to the antibacterial compound’s impact on infection progression, its effect on bone integration is also important. To investigate bone integration, the study duration should be sufficient for bone to remodel. The studies included in this review that investigated bone growth around the implant most frequently used 42 days (*n* = 7) (Figure 5a). Several of these studies concluded that six weeks was sufficient for bone apposition on the implant surfaces [72,78,98,115,127]. Efstathopoulos et al. [80] studied bone growth from two to six weeks after implant removal and insertion of enriched acrylic bone cement, and they concluded that bone remodeling was best at six weeks. Several other studies, not included in this review, that investigated bone integration of an implant found that bone formation starts at three weeks, and a rigid bone–implant interface is seen at six weeks [128,129,130,131,132]. Hermida et al. [133] concluded there was no significant difference in bone-to-implant contact between six and twelve weeks. These studies suggest that six weeks is likely sufficient to assess the osseointegration of the implant and the effect of the antibacterial compound on this integration.

#### 4.2.2. Inoculum—Bacterial Strain

Of the included studies in this research, 90% used one or multiple strains of *S. aureus* to inoculate the rabbits (Figure 5b). Again, the pathogenic factor needs to be chosen depending on the aim of the study. As previously stated, most PJIs are caused by *S. aureus* and *S. epidermidis* [7,9]. While *S. aureus* is the most common pathogen in Europe and China, *S. epidermidis* is most common in the US [15,134]. Therefore, depending on the location of the study, different pathogens might be chosen. Although *S. aureus* and *S. epidermidis* are the most common pathogens in PJI, Gahukamble et al. [70] focused on *Staphylococcus lugdunensis* and *Propionibacterium acnes*, pathogens that used to be seen as contaminants when culturing clinical isolates. As *S. lugdunensis* and *P. acnes* are increasingly recognized as pathogens causing PJI, they state the importance of observing these bacteria in a rabbit model [135,136,137]. Few studies focus on Klebsiella pneumonia and *P. aeruginosa*, though PJI involving these pathogens is difficult to treat and requires more research [81,103,138]. As *P. aeruginosa* often shows in polymicrobial infections with *S. aureus*, Bitika et al. [103] inoculated with both bacteria. The World Health Organization has published a list of priority pathogens that require new antibacterial techniques. This list emphasizes the importance of expanding research beyond *S. aureus* alone [139].

Although 90% of all included studies in this review use *S. aureus* to inoculate the rabbits, 29 different strains of *S. aureus* have been used. These 29 strains range from standard cell lines to clinical isolates (33%), and from MRSA (28%) to MSSA (specifically stated for 12%). However, studies often do not state if their strain is a clinical isolate (66%) or if they use MRSA or MSSA (50%). The strain can impact the study outcomes, as each strain can exhibit significantly different characteristics. The strains can differ in toxin production, biofilm formation, gene regulator types, immune evasion mechanisms, and the possibility of creating persister cells or small colony variants [140]. Tuchscherr et al. [141] found a wide range of cytotoxicity and invasiveness between multiple clinical *S. aureus* strains. Interestingly, they also found that the host cleared low-cytotoxicity strains less efficiently compared to the highly cytotoxic strains [141]. Guo et al. [142] investigated 20 cohort studies on PJI and determined that MSSA was responsible for PJI at a rate 2.5 times higher than that caused by MRSA. However, the chance of treatment failure is higher for MRSA compared to MSSA [143,144]. Understanding the characteristics of the bacterial strain is important, as all these factors can influence their susceptibility to the tested antibacterial technologies. Though clinical isolates or resistant strains have greater clinical relevance for research, standard cell lines have the advantage of being better characterized and provide a more standardized result.

#### 4.2.3. Inoculum—Dose

Inoculum sizes in the included studies in this research ranged from 4.3 × 10^1^ CFU [101] to 6 × 10^8^ CFU [94] (Figure 5c). This range of inoculum doses makes it extremely difficult to compare infection clearance rates between experiments and antibacterial technologies. When choosing the inoculum size, it is important to ensure that the CFU concentration in the inoculum is sufficient to establish an infection in the untreated rabbits that their immune systems cannot clear, while avoiding an excessively high and clinically unrealistic CFU concentration. Of the 20% of studies that used no antibacterial intervention, several studies were dose-finding studies for the number of CFUs needed to create a PJI. Craig et al. [69] found that a lower incidence of infection occurred with a low dose of 10^2^ CFU compared to higher doses. They also state that inoculums >10^6^ CFU will probably lead to greater animal morbidity due to sepsis [69]. This was confirmed by Poultsides et al. [73], who found a 100% dropout rate in the group that received 5 × 10^8^ CFU. The appropriate inoculum dose should be selected based on the study aim, the chosen strain, and its virulence.

#### 4.2.4. Inoculum—Administration

Besides differences in bacterial strains and inoculum sizes, the administration method of the inoculum is another variable. There are several administration routes that can take place prior to or after the insertion of the implant, and they can be contained or not. The influence of these administration methods is often overlooked. PJI can arise in a patient due to several causes, and the main contributor is still under debate. Contamination can arise during surgery; from hematogenous spread from infections elsewhere in the body or intravascular devices; from direct extension from the skin; from a bacterial reservoir in the deeper skin; or from soft tissue close to the implant [145,146,147]. Zeller et al. [148] classified clinical PJIs as hematogenous (35%), late chronic (30%), early postoperative (19%), or undetermined (16%). The studies included in this review used several methods to create an infection in the rabbits, as can be seen in Figure 5d. Most studies inoculated the subjects by injecting the bacteria into the medullary cavity in which the implant was inserted (45.0%). Others were inoculated by injecting bacteria into the joint (23.3%), via intra-articular injection (13.3%), by incubating the implant in a bacterial inoculum before insertion (11.7%), or by injection in the bone defect (1.7%). A total of 5% of the studies did not state the method of inoculation. Of the included studies, 78.3% did not employ a method to contain the inoculum in the insertion site, 16.7% used bone wax, and 5.0% used other options to contain the inoculum. As there is no one way for patients to contract PJI, there is also no one way to induce it in vivo.

#### 4.2.5. Implant

Whereas the femur is the largest long bone that can be implanted, the tibia is more easily accessible [33]. The most popular choices of bone in the included studies in this research are therefore the tibia (51.7%), the femur (38.3%), or a combination of the two (3.3%) (Figure 5e). Only 1.7% used the humerus, and 5.0% used the radius. This variability is also in alignment with the distribution of arthroplasties in patients. Currently, TKAs and THAs extremely outnumber TSA. However, the incidence of TSA is increasing faster than that of TKAs and THAs, increasing the need for research on the humerus [149]. Most implants are a simplification of implants used in humans; therefore, the intended use is not completely mimicked, and which long bone is used in the rabbits is less critical. Again, it is important to look at what outcome measures are studied. In a study that only looks at antimicrobial activity, the choice of bone is of less importance compared to when osseointegration is also studied, as in these cases the weight-bearing qualities of the limb can affect the results. As rabbits jump off with their hind legs, the femur and tibia experience more force than the radius and humerus. This difference in movement mechanics should also be considered when looking at bone ingrowth.

#### 4.2.6. Experimental Groups and Group Size

The number of experimental groups and group size differ greatly between all studies included in this review. The number of experimental groups ranges from only one group to testing and comparing several antibacterial technologies, testing both infected and uninfected controls, testing several inoculum sizes and time points on one antibacterial technology, and testing several bacterial strains. Especially in the studies that did not test an intervention, but aimed to establish and validate a new model, uninfected rabbits were used as controls. An uncoated implant or the use of the current standard of care with an infection was used as a control in most other studies. López-Torres et al. [71] had only one study group. However, they aimed to set up and validate the PJI rabbit model without the use of any intervention. Three other studies used one limb as the control group and one limb as the experimental group within the same rabbit [87,103,118]. Craig et al. [69] also tested several inoculum sizes or saline as control within the same rabbit. Using one rabbit for the control and experimental group may create experimental inaccuracy, as the infection is not restricted to the limb it is injected into. If systemic infection develops, or a rabbit reaches a humane endpoint, it is unknown to which group this belongs. Brunotte et al. [79] tested four different inoculum sizes in their rabbits but did not have a control group. Having a control group that is either uninfected or has no antibacterial technology is important to set a baseline to which to compare the intervention groups. Furthermore, a control group validates the model, as it ensures that observed effects in the other groups are due to the intervention. Concerning group sizes, López-Torres et al. [71] use the largest group size, with fifteen rabbits in one experimental group. On the contrary, Moriarty et al. [101] and Horn et al. [100] tested different inoculum sizes per experimental group, resulting in subgroup sizes of only one rabbit. Yang et al. [112] had the smallest (full) group size, with three rabbits per group. Zhao et al. [115] and Zhou et al. [117] did not report on the total number of rabbits used, or the experimental group size, making their results unreliable. To increase the validity, accuracy, and reliability of the experimental results, a power calculation should be conducted to estimate the experimental group size [24,48,150]. Applying this power calculation reduces the unnecessary use of rabbits. As underpowered experiments produce unreliable results and overpowered experiments use more animals than necessary, it is a delicate balance [48]. Predicting the effect size in a power calculation might be challenging because of the lack of correlation between in vitro and in vivo data.

#### 4.2.7. Dropout Number

In 40% of the studies included in this research, the dropout number was not stated, or the total number of rabbits per group that survived could not be found in the results. This does not follow the ARRIVE guidelines, as they state that any adverse outcome should be described [43,44]. For the studies that reported on dropout numbers, seven studies did not specify to which group the dropped-out rabbits belonged [79,83,100,101,111,119,126]. Furthermore, four studies replaced the dropped-out rabbits with new rabbits [94,100,106,111]. Two of these studies, by Horn et al. [100] and Oosterbos et al. [111], did not state which group the replaced animals belonged to. Replacing these animals raises data integrity concerns as data consistency and reliability are compromised. Even if a full study duration is not completed, the data created by this rabbit’s response are valuable. Studies are meticulously designed with specific sample sizes and conditions. Introducing new subjects midway can introduce variables that were not accounted for initially, such as differences in age, weight, and health status. Furthermore, blinding and randomization are not possible anymore when rabbits from only specific groups are replaced. Especially in the studies where it is not mentioned which animals are replaced, or where animals from the group with the antibacterial compound are replaced, bias is introduced. This bias and variability can potentially skew the results toward a better outcome for a desired group, creating difficulty in drawing conclusions. Furthermore, replacing these animals raises ethical concerns. It creates a disregard for individual animal welfare and is not in line with Russel and Burch’s 3Rs [38]. Replacing dropped-out rabbits often means subjecting additional animals to potential pain, stress, and suffering associated with experimental procedures. Studies should always report on their dropout number, and refrain from replacing rabbits. Instead, they should design their studies with appropriate sample sizes that account for potential dropouts. Furthermore, statistical methods are available to handle missing data.

**Table 2:** Experimental design extraction results—part 1. Abbreviations used: ns = not stated.

References	Aim of Study	Duration of the Study (Days)	Inoculum			
			Strain (c.i. = Clinical Isolate)	Inoculum Size (Absolute CFU/Used Volume)	Administration Way/Site	Containment Method
**No intervention used**						
[69]	To design and evaluate a novel small animal model for the investigation of biomaterial centered infection in total joint arthroplasty with future plans to utilize this model for the evaluation of novel anti-infective therapeutics.	7	MRSA ST-021 (c.i.)	100 µL saline, 10^2^, 10^3^ or 10^4^ CFU in 100 µL saline in other knee.	Injected into knee joint	None
[70]	To observe the effects of *P. acnes* and *S. lugdunensis* in an established rabbit model of implant-related osteomyelitis, in the absence of implant material wear debris, and characterize the resultant infections with respect to histological and microbiological outcomes.	2	*P. acnes*, LED2, and *S. lugdunensis* 010729 (all c.i.)	3 × 10^7^ CFU in 50 µL	Injected into the tibial medullary cavity	Water-soluble alkylene copolymer
[71]	To develop an animal model which is clinically representative of PJI and can serve as a foundation for future research to develop new therapeutic and preventative strategies.	7	*S. aureus* ATCC 29213	10^5^ CFU in 1 mL	Intra-articular inoculation with 25-G needle	None
[29]	To establish an improved rabbit implant infection model, based on several previously published models resembling clinical orthopaedic implant infections.	42	MSSA UAMS-1 ATCC 49230 (c.i.)	3.8 × 10^5^ CFU in 100 µL saline	Injected intramedullary	Bone wax
[72]	To evaluate 18F-FDG microPET as an implant osteomyelitis imaging tool using a *Staphylococcus aureus*-induced peroperative implant infection in rabbits.	42	*S. aureus* UAMS-1 ATCC 49230	3.8 × 10^4^ CFU in 100 µL saline	Injected into intramedullary cavity	Bone wax
[73]	The aim of this study is to establish a new experimental model of hematogenous implant-related infection (IRI) by a community-acquired methicillin-resistant *S. aureus* (CA-MRSA) strain.	56	MRSA MLST-80 clone	3 or 5 × 10^8^ CFU in 1 mL saline	Injection with 26-G catheter through femoral artery	None
[74]	To test the ability of 99mTc-ciprofloxacin imaging to discriminate between infected and uninfected prosthetic joints, using a previously validated rabbit model of prosthetic joint infection.	20	MSSA (c.i.)	10^7^ CFU/0.5 mL	Intra-articular injection	None
[75]	To test the ability of 99mTc-UBI 29-41 (UBI) to discriminate between infected and uninfected prosthetic joints using a previously validated rabbit model.	20	MSSA 17548 (c.i.)	10^7^ CFU/0.5 mL	Intra-articular injection	None
[76]	To investigate whether 99mTc-annexin V imaging was effective in differentiating early stage PJI from uninfected prosthetic joints in a validated rabbit model.	21	MSSA ATCC29213	10^6^ CFU/0.5 mL	Injected with a 1 mL syringe at the level of the lower patellar border	None
[77]	To establish and evaluate a novel PJI animal model with different bacterial concentrations using 18F-fluorodeoxyglucose (FDG) and 68Ga-fibroblast activation protein inhibitor (FAPI) PET/CT.	14	*S. aureus* ATCC29213	10^7^ CFU, 10^6^ CFU, 10^5^ CFU and 10^4^ CFU/0.5 mL saline	Injected in canal	None
[78]	To explore the performance characteristics of 18F-FDG and 68Ga-FAPI in PJI and aseptic loosening models.	42	ns	Control group no inoculum, 10^5^ CFU *S. aureus*, 10^8^ CFU *S. epidermidis*/0.5 mL	Injected into knee joint	None
[30]	The purpose of this study was to develop and characterize a rabbit model of chronic PJI using common radiological and clinical markers.	28	*S. aureus* ATCC 25923	1 × 10^6^ CFU/1 µL	Intraosseous injection into a predrilled bone tunnel	Bone wax
**Revision**						
[79]	The aim of this study was to establish a new small animal model for simulating a two-stage-revision procedure by implant-related MRSA infections.	84	MRSA, EDCC 5443, and EDCC 5398, (all c.i.)	10^5^ or 10^7^ CFU in 20 µL	Intramedullary tibia canal injection	None
[80]	To evaluated the efficacy of a system designed to gradually release grepafloxacin from acrylic bone cement (polymethyl methacrylate, PMMA) for the treatment of experimentally chronic osteomyelitis induced in rabbits.	35–63	MRSA (c.i.)	1 × 10^7^ CFU/100 µL	Injection into upper third of the right femur	None
[81]	To evaluate the efficacy of a colistin-impregnated cement spacer, alone or in combination with systemic colistin with or without systemic meropenem, using a rabbit model of PJI caused by carbapenemase-producing Klebsiella pneumoniae (CPKP) that closely mimics human infection, adapted from a previous model.	14	KPC-producing Klebsiella pneumonia KPC99Y (c.i.)	5 × 10^8^ CFU in 0.5 mL	Injected into knee, close to prosthesis	None
[82]	To compared the efficacy of a teicoplanin-impregnated cement spacer alone with that of a teicoplanin-impregnated cement spacer combined with systemic teicoplanin, using a rabbit model of MRSA knee prosthesis infection that closely mimics human infection.	28	MRSA	10^8^ CFU in 0.5 mL	Injection into knee, close to prosthesis	None
[83]	To determine the effectiveness of bone cement containing rifampicin microcapsules, to establish the in vivo safety profile of microencapsulated rifampicin and to register the rifampicin intra-articular release profile.	28	MSSA, ATCC29213	10^5^ CFU in 1 mL	Intra-articular injection	None
[84]	To compare the efficacy of tobramycin-containing bone cement with that of systemic cefazolin for treatment of infection in a one-stage revision model.	42	*S. aureus* Wood-46, ATCC 10832	10^6^ CFU in 0.1 mL or 10^5^ CFU in 0.1 mL	Injected into medullary canal	None
[85]	To study the effectiveness of gentamicin-loaded PNDJ (G-PNDJ) hydrogels in treating orthopaedic infections in a rabbit model.	49	*S. aureus* UAMS-1, ATCC 4923	7.5 × 10^6^ CFU in 50 µL TSB	Inoculated after wire implantation, unspecified how	None
[86]	To test the effect of continuous wave ultrasound (CWU) on antimicrobial efficacy of antibiotic-loaded bone cement (ALBC) assessed by clinical performance, radiology, bacteriology, and histology in vivo in a rabbit model.	60	*S. aureus* ATCC13565	10^8^ CFU in 0.1 mL	Injected into upper femur and joint cavity	Surgical wax
**Prevention: antibiotics only**						
[87]	To assess the effectiveness of tobramycin-impregnated microspheres in preventing infection, and to analyse if implant integration was affected by the presence of infection or the microspheres themselves.	14	*S. aureus* ATCC 49230	20 × 10^6^ CFU in 10 µL	Pipetted directly onto the implant	None
[88]	This study examined and compared the antimicrobial effectiveness of teicoplanin- and clindamycin-coated titanium wires on an experimental model of *Staphylococcus aureus* infection.	7	*S. aureus* ATCC 29123	500 CFU, volume unknown	21G green needle in intramedullary canal	None
[89]	The objective of this animal study was to examine the efficacy of a coating of minocycline and rifampin to prevent colonization of a grit-blasted titanium-alloy implant, osteomyelitis, and device-related osteomyelitis due to *S. aureus*.	7	*S. aureus* ATCC25923	0.5 × 10^3^ CFU/mL in 25 µL	Injected in intramedullary canal of femur	None
[90]	To compare the efficacies of ceftaroline-fosamil (CPT-F) or vancomycin (VAN) alone or combined with rifampin (RIF) against MRSA in a knee PJI model in rabbits that closely simulates human infection.	17	MRSA ST20121238 (c.i.)	5 × 10^7^ CFU in 0.5 mL	Injected close to prosthesis after closing the skin	None
[91]	To test the ability of an antibacterial-loaded bioreabsorbable hydrogel coating (DAC^®^), obtained by derivatization of low molecular weight hyaluronic acid (HA) with poly-D,L-lactic acid (PDLLA), to reduce bacterial acute colonization in an animal model of intraoperative high-load bacterial contamination of an implant.	7 or 84 *^1^	MRSA (c.i.)	10^6^ or 10^4^ CFU in 0.2 mL	Injected into medullary cavity with an 18-gauge needle	Bone wax
[92]	To establish a new MRSA peri-implant osteomyelitis animal model, and to determine clinical parameters to monitor the infection after MRSA inoculation, and under anti-microbiological therapy with vancomycin.	4	MRSA ATCC33591	10^6^ CFU in 25 μL	Coated intra-operatively on implant	None
[93]	To evaluate the surface morphologies, hydrogel swelling, drug release kinetics and antibacterial properties of a localized drug delivery system.	7, 14, 28	*S. aureus* ATCC29213	3 × 10^7^ CFU/0.1 mL	Injected into marrow cavity	Bone wax
[94]	To investigate using a coprecipitation drug-loading approach the effects of nanotubular anodized titanium coated with gentamicin (NTATi-G) on infection prevention and bone cell biocompatibility in a rabbit model with *Staphylococcus aureus* inoculation in the tibial metaphysis.	42	*S. aureus* ATCC25923	6 × 10^8^ CFUin 0.2 mL	Injected into intramedullary canal	Bone wax
[95]	To evaluate the efficacy of levofloxacin, alone or in combination with rifampin, for treatment of rabbit experimental prosthetic knee infections due to *S. aureus*	17	*S. aureus* 17848 (c.i)	10^7^ CFU/0.5 mL PBS	Injected into closed knee, close to the prosthesis	None
[96]	To compare the efficacies of quinupristin-dalfopristin (Q-D) and vancomycin, alone and in combination with rifampin, using a rabbit model of experimental MRSA knee prosthesis infection that closely mimics MRSA infections in humans.	14	MRSA strain HM 1054 (c.i.)	5 × 10^7^ CFU/0.5 mL	Injection close to prosthesis after surgery	None
[97]	To compare the efficacie of of high-dose daptomycin (equivalent to 8 mg/kg/day in humans) or vancomycin, both alone and with adjunctive rifampin, in an experimental MRSA joint prosthesis infection.	17	MRSA S271 (c.i.)	5 × 10^7^ CFU in 0.5 mL PBS	Injected in knee close to prosthesis after surgery	None
[98]	To compare linezolid and vancomycin in the treatment of MRSA infections of orthopedic implants, in a new rabbit model with titanium implants experimentally infected with MRSA.	42	MRSA ATCC33591	10^6^ CFU/25 µL	Implants coated before surgery	None
**Prevention: surface modification**						
[99]	To evaluate the antibiotic release, in vitro cytocompatibility, and in vivo effectiveness in preventing PJI caused by *S. aureus* of the F- and P-doped, bottle-shaped nanotubular oxide layer grown in Ti-6Al-4V alloy loaded with a mixture of gentamicin and vancomycin (GV).	35	*S. aureus* Sa5 (c.i.)	10^6^ CFU in 100 μL	Injected into medullary canal through the intertrochanteric crest	None
[100]	The aim of our study was to determine if the local resistance to infection of a cannulated IM nail is less than that of a solid nail and more similar to that of a hollow nail.	28	*S. aureus* V 8189-94 (c.i.)	4 × 10^4^ to 4 × 10^6^ CFU/100 μL	Intravenous catheter into medullary cavity	Hemostatic collagen plug
[101]	The aim of the present study was to determine the effect of polishing TAN IM nails on susceptibility to infection in an animal model.	28	*S. aureus*, JAR 06.01.31 (c.i.)	4.3 × 10^1^ CFU, 4.3 × 10^2^, 4.3 × 10^3^ or 4.3 × 10^4^ CFU in 50 µL	Injected into medullary canal with 14 gauge catheter	Water soluble alkaline co-polymer
[102]	To evaluate in an in vivo normal model the osteogenic response and the osteointegration of an anodic spark deposition nanostructured titanium surface doped with gallium (ASD + Ga) in comparison with two other surface treatments of titanium: an anodic spark deposition treatment without gallium (ASD) and an acid etching treatment (CTR). Moreover the study assesses the osteoprotective potential and the antibacterial effect of the previously mentioned surface treatments in an experimentally-induced peri-implantitis model.	7 or 14	*S. aureus* ATCC 25923	10^5^ CFU/mL, volume unknown	Implant inoculated for 6 min before placement	None
**Prevention: coating**						
[103]	To test the antibacterial efficacy of silver coated titanium implants in an in vivo contaminated rabbit knee fixation model before proceeding with clinical studies.	84	*S. aureus*, *P. aeruginosa* (all c.i.)	2 × 10^3^ CFU/0.2 mL for *S. aureus*, 2 × 10^7^ CFU/0.2 mL for *P. aeruginosa*	Injection into the joint capsule using a 30 G needle and 0.5 mL syringe.	None
[104]	To investigate the hyaluronic-acid-based hydrogel DAC^®^ as carrier for local delivery of antimicrobial agents for infection in an in vivo implant-related infection model.	28	*S. aureus* Wood 46 ATCC 10832	10^5^ CFU in 50 µL	Injected in medullary canal before placing implant	None
[105]	First, to establish a suitable in vivo osteomyelitis model in rabbits, second to evaluate the antimicrobial activity of a silver multilayer coating (SML) under realistic pre-clinical conditions.	7	MSSE RKI 10-0062 (c.i.)	2 × 10^4^ CFU, volume ns	Wires incubated in a test tube over a length of 9 cm in bacterial solution for 30 to 60 min under dynamic conditions, with an inoculum of ~1 × 10^6^ CFU/mL	None
[106]	To test whether silver ion-containing calcium phosphate-based ceramic nanopowder-coated implants prevented implant-related infection by comparing silver-coated, hydroxyapatite (HA)-coated, and uncoated titanium implants in vivo using radiology, histology, and microbiology	42	MRSA ATCC43300	5 × 10^2^ CFU/50 μL	Injected with small pipette into medullary canal	None
[107]	To test if silver ion doped calcium phosphate based ceramic nano-powder coated intramedullary nails prevent bacterial infection as compared with uncoated nails in an in vivo rabbit study.	70	MRSA ATCC 43300	0.5 × 10^5^ CFU in 50 µL	Injected into intramedullary canal	None
[108]	To study the efficacy of a biodegradable Polymer-Lipid Encapsulation MatriX (PLEX) loaded with the antibiotic doxycycline as a local prophylactic strategy against implant-associated osteomyelitis. Activity was tested against both a doxycycline-susceptible (doxyS) methicillin-susceptible *S. aureus* (MSSA) as well as a doxycycline-resistant (doxyR) methicillin-resistant *S. aureus* (MRSA).	28	*S. aureus* JAR060131 (doxyS MSSA) MRSA strain LUH15101 (doxyR MRSA), (all c.i.)	DoxyS: 5.9 ± 1.3 × 10^4^ CFU/100 μL; DoxyR: 4.7 ± 1.2 × 10^5^ CFU/100 μL	Pipetted into medullary cavity	None
[109]	To investigate in an in vivo contaminated implant bed model, the efficacy of adding tobramycin to a PA-coated titanium foam implant in preventing implant related Staphylococcal infection and study the effects on osseointegration; this in comparison to both PA-coated and uncoated implants.	28	*S. aureus* Wood 46, ATCC10832	None, 10^3^, 10^4^ or 10^5^ CFU/100 μL	Injected with micropipette into medullary canal	None
[110]	This study describes the development of a new, robust hydroxyapatite (HA)-coating containing gentamicin on titanium alloy (Ti-6Al-4V) covered with a protective, biodegradable poly (lactic-co-glycolic acid) (PLGA)-overlayer, that prevents colonization of cementless orthopaedic prostheses by perioperatively introduced bacteria. In vivo evaluation of the antibacterial efficacy of the PLGA-gentamicin-HA-coating was carried out in a contaminated prosthesis model in rabbits, while effects of the coating on bone fixation and osseointegration were assessed in a canine condylar defect model, to demonstrate technology potential for clinical translation.	2 or 7	*S. aureus* ATCC 25923	1 × 10^5^ CFU/100 µL	Pipetted into medullary canal	Bone wax
[111]	To investigate histomorphometrically the osseointegration (bone contact and bone area) of hydroxyapatite (HA)-coated and noncoated titanium implants in the presence of local infection compared with the absence of local infection.	28	*S. aureus* Wood 46 ATCC 10832	10^2^, 10^3^, 10^4^, or 10^5^ CFU/0.1 mL	Pipetted in medullary canal	None
[112]	In this study, the post porous hydroxyapatite (HA) coated Ti4Al4V is prepared for the subsequent electrolytic deposition of vancomycin–chitosan composite to control the drug release. The aim of this study is to test the antibacterial effect in a rabbit infection animal model.	28	*S. aureus* ATCC 6538P	10^7^ CFU/20 µL	Injected with 16G needle before insertion pin	None
[113]	In this study, they created Ag-supported/TiO_2_ nanotubes (Ag/TNT) by a combination of electrochemical anodization and pulse electrodeposition and prepare a super-hydrophobic coating by modifying the surface of the Ag/TiO_2_ nanotubes with 1H, 1H, 2H, 2H-perfluorooctyl-triethoxysilane (PTES). We evaluate the inhibitory effect of the coating on bacterial adhesion and killing adherent bacteria and assess its effect on Ag release.	28	*S. aureus* 8325	2 × 10^2^ CFU/20 µL	Injected into medullary cavity	Bone wax
[114]	The aims of the present study were to: (1) characterize the relation between Cu2+ dose and antibacterial activity and in vitro biocompatibility; (2) test the effect of the coating in a small animal bone fracture model	28	*S. aureus* BNCC186335	10^4^ CFU/100 µL	Injected into proximal and distal parts of tibia after transection in the middle	None
[115]	The antibacterial efficacy and osteogenic properties of ZnO and ZnO/Zn_3_(PO_4_)_2_ nanostructures on Ti-based implants are systematically evaluated in vitro and in vivo, and the underlying mechanisms are carefully dissected.	14 or 42 *^2^	*S. aureus*	10^5^ CFU/mL, volume unknown	Implants were incubated for 30 min before implantation	None
[116]	In the present study, biomimetic hierarchical micropore/nanorod patterned coatings (MNRs) on Ti were developed, in which the nanorods revealed a fixed interrod spacing of about 70 nm and consisted of fluorine (F) incorporated Ca9Sr1(PO4)6(OH)2 (Sr1−HA, strontium containing hydroxyapatite) with the fixed Sr but different F content. The antibacterial activities were assessed in a bacterial-infected rabbit model.	56	*S. aureus* ATCC43300	2 × 10^3^ CFU/20 µL	Injected into medullary cavity with a microsyringe	None
[117]	The aim was to test titanium as implant covalently immobilized with a polyethylene glycol (PEG)-based thermoresponsive polymer (MPEG) and an antimicrobial peptide (AMP) HHC36 onto the implant surface. We demonstrated that the two components endowed the surface with spatiotemporal control over the different biofunctions at the three service stages of the implant. The in vivo behavior in two infection models in New Zealand white rabbits was tested.	7 and 60	*S. aureus* ATCC 6538P	7.5 × 10^6^ CFU/15 µL	ns	None
**Other**						
[118]	To test if a dilute Betadine lavage of 3.5% would achieve a significant decrease in bacterial counts compared with an isolated saline lavage in an in vivo knee PJI model.	14	*S. aureus* ATCC 25923	10^6^ CFU/100 µL	Injected with 22-gauge needle in knee joint	None
[119]	To investigate biodistribution and tolerability of oly(*n*-isopropylacrylamide-co-dimethylbutyrolactone acrylamide-co-Jeffamine M-1000 acrylamide) (PNDJ) hydrogels as sustained release carriers.	49	MSSA ATCC 49230, MRSA ATCC BAA-1556	7.5 × 10^6^ CFU/volume ns	Administered before closing in defect radius	None
[120]	To evaluated the late resistance to hematogenous contamination by microbial pathogens of implants and bone-implant interface and the development of late clinical infection when cementless components with different surface or structural properties are implanted	56	MRSA strainMLST-80 PVL+ clone (c.i.)	3 × 10^8^ CFU/1 mL (group B-E) or 1 mL sterile saline (group A)	Injected with 26-gauge needle in femoral artery 4 weeks after implantation	None
[121]	To evaluate the efficacy of a bioabsorbable antibiotic containing bone (Ab-PLGA) screw compared to a stainless steel (SS) screw in the prevention of biomaterial-related infection due to *Staphylococcus aureus*.	42	*S. aureus* 52/52A/80	3 × 10^4^ CFU/mL, volume unknown	6 min incubation of screw before implantation	None
[122]	A rabbit PJI model was used with the highly pathogenic USA300 community-associated methicillin-resistant *S. aureus* (MRSA) strain to further evaluate the protective efficacy of a combination of three previously described monoclonal antibodies (MAbs) targeting alpha-hemolysin (Hla) with MEDI4893 *, clumping factor A with AZD7745 *, and leukocidins (LukSF, LukED, HlgAB, and HlgBC) with AZD8887 (AZD6389 *).	8	MRSA USA300/SF8300	5 × 10^5^ CFU/300 μL	Intra-articular injection	None
[123]	In present study, in order to determine the antibacterial activity of the new Ti–Cu sintered alloy we conducted an in vivo experiment on the basis of previous in vitro research by Zhang et al.	14	*S. aureus* ATCC6538	1.0 × 10^5^ CFU/10 µL	Injected into medullary cavity	Bone wax
[124]	The aim of this study was to investigate the effects of allicin on biofilm formation, and whether allicin could potentiate the bactericidal effect of vancomycin in a rabbit PJI model.	17	*S. epidermidis* RP62A	10^4^ CFU in 1 mL	Injected into knee joint	None
[125]	The present study aimed to isolate broad-range bacteriocins from Lactobacillus rhamnosus (ATCC 53103) and investigate their antibacterial effect on *S. aureus* in a rabbit model of knee implant infection.	5	*S. aureus* ATCC29213	1.5 × 10^5^ CFU/0.5 mL	Intra-articular injection	None
[126]	The purpose of the experiment is to verify the preventive effect of antibacterial peptide PR39 on periprosthetic infection, which may provide a new solution for the treatment of periprosthetic infection in the future.	14	*S. aureus* ATCC 25923	4 × 10^6^ CFU/100 µL	Injected into knee joint	None

*^1^ 7 days for treatment groups, 84 days for histocompatibility evaluation; ^*2^ 14 days for antibacterial properties, 42 days for osseointegration.

**Table 3:** Experimental design extraction results—part 2. Abbreviations used: Impl. = implant; VAN = vancomicin; s.s. = stainless steel; Ti = titanium; GEN = Gentamicin; RIF = Rifampicin; ns = not stated.

References	Implant				Interventions Used against PJI	Total Number of Rabbits	Experimental Groups and Number of Rabbits per Group (*n*)	Dropout Number	
	Description	Material	Movement Prevention	Location (Specified)				Per Experimental Group [%]	Total [%]
**No intervention used**									
[69]	Diameter 4.0 mm, length 15 mm	S.S. cannulated screw with sterile UHMWPE washer	PMMA	Femur (lateral femoral condyle)	None	22	100 µL saline in one knee, 10^4^ CFU in 100 µL saline in other knee (*n* = 11)	9	5
							10^2^ in 100 µL saline in one knee, 10^3^ in 100 µL saline in other knee (*n* = 11)	0	
[70]	Diameter 2.5 mm, length 85 m	S.S.	No	Tibia (medullary cavity)	None	18	Uninoculated (*n* = 6)	0	0
							*P. acnes* (*n* = 6)	0	
							*S. lugdumensis* (*n* = 6)	0	
[71]	Tibial insert, ‘rabbit specific’ implants made using CT scans and 3D reconstruction, mimicking anatomical irregularities of the tibial plateau	S.S.	Metaphyseal anchoring and bone cement	Tibia (implant replaced the tibial plateau surface)	None	15	Tibial insert (*n* = 15)	0	0
[29]	Length 20 mm, diameter 4 mm	Grit-blasted Ti (TiAl6V4)	No	Tibia (proximal medullary cavity)	None	22	Contamination group (*n* = 11)	18	27
							Sterile saline control group (*n* = 11)	36	
[72]	Length 20 mm, diameter 4 mm	Grit-blasted Ti alloy (TiAl6V4)	No	Tibia (proximal medullary cavity)	None	22	Uncontaminated implant group (*n* = 11)	27	32
							Contaminated implant group (*n* = 11)	36	
[73]	Cylinder diameter 3 mm, length 30 mm, cylindrical cup 5 × 5 mm	Porous tantalum, cylindrical silicon cup	No	Tibia (proximal medullary canal)	None	30	Received 1 mL of 5 × 10^8^ CFU/mL at 4 weeks (*n* = 10)	100	33
							Received 1 mL of 3 × 10^8^ CFU/mL at 4 weeks (*n* = 10)	0	
							Received 1 mL saline at 4 weeks (*n* = 10)	0	
[74]	Tibial component (Silastic, great toe implant HP, Swanson Design; Dow Corning, Valbonne, France), implant head 15 × 5 mm, stem length 14 mm	Silicone elastomer	No	Tibia (tibial plateau and medullary cavity of the metaphysis)	None	13	Infected (*n* = 6)	33	23
							Uninfected (*n* = 7)	14	
[75]	Tibial component (Silastic, great toe implant HP, Swanson Design; Dow Corning, Valbonne, France), implant head 15 × 5 mm, stem length 14 mm	Silicone elastomer	No	Tibia (tibial plateau and medullary cavity of the metaphysis)	None	20	Infected (*n* = 12)		ns
							Uninfected, 1 mL saline injected (*n* = 8)		
[76]	Tibial component (Silastic, great toe implant HP, Swanson Design; Dow Corning, Valbonne, France), implant head 15 × 5 mm, stem length 14 mm	Silicon elastomer	No	Tibia (tibial plateau and medullary cavity of the metaphysis)	None	24	Infected (*n* = 12)		ns
							Uninfected, injection of 0.5 mL saline (*n* = 12)		
[77]	Screw with diameter 4 mm, length 20 mm	ns	Self-locking	Femur and tibia (in femoral shaft at intercondylar notch and ACL footprint in tibia)	None	40	0.5 mL saline (*n* = 8)	12	23
							2 × 10^7^ CFU/mL in 0.5 mL saline (*n* = 8)	38	
							2 × 10^6^ CFU/mL in 0.5 mL saline (*n* = 8)	13	
							2 × 10^5^ CFU/mL in 0.5 mL saline (*n* = 8)	38	
							2 × 10^4^ CFU/mL in 0.5 ml saline (*n* = 8)	13	
[78]	Screws, diameter 3 mm, length 20 mm	ns	Self-locking	Femur (intercondylar fossa and anterior cruciate ligament	None	36	Control (*n* = 6)	0	19
							Aseptic loosening (*n* = 10)	20	
							*S. aureus* (*n* = 10)	30	
							*S. epidermidis* (*n* = 10)	20	
[30]	Screw	ns	ns	Femur (distal femur knee joint)	None	13	Infected (*n* = 5)	0	0
							Non-infected (*n* = 5)	0	
							Separate cohort (*n* = 3) for bacterial viability assays	0	
**Revision**									
[79]	ns	K-wires, S.S.	No	Tibia (medullary canal)	Two-stage revision with debridement + VAN loaded cement spacers. A total of 1.2 g of VAN was used per 40 g of PMMA. The local VAN release rate was 1.569 mg VAN over four days.	12	MRSA EDCC 5443, 10^5^ CFUs (*n* = 3)	ns	8
							MRSA EDCC 5443, 10^7^ CFUs (*n* = 3)	ns	
							MRSA EDCC 5398, 10^5^ CFUs (*n* = 3)	ns	
							MRSA EDCC 5398, 10^7^ CFUs (*n* = 3)	ns	
[80]	ns	Metal needle	No	Femur (upper third of the right femur)	After 3 weeks, needle/implant is removed. Followed by a local injection of a mixture of acrylic bone cement enriched by grepafloxacin 4%. Per time point 1 control animal.	36	Sacrificed at week 2 (*n* = 6)		ns
							Sacrificed at week 3 (*n* = 6)		
							Sacrificed at week 4 (*n* = 6)		
							Sacrificed at week 5 (*n* = 6)		
							Sacrificed at week 6 (*n* = 6)		
[81]	Arthroplasty implant of the first metatarsophalangeal joint (Silastic, great toe implant HP; Swanson Design, Dow-Cornin)	Silicone elastomer	No	Tibia (partial knee replacement with tibial component)	Seven days after inoculation the prosthesis was removed and replaced by a cement spacer. Six treatment/control groups: (i) drug-free spacer; (ii) colistin-loaded spacer; (iii) drug-free spacer + colistin intramuscular (i.m.); (iv) colistin i.m. + colistin spacer; (v) drug-free spacer + colistin i.m. + meropenem subcutaneous (s.c.); and (vi) colistin i.m. + meropenem s.c. + colistin spacer.	72	Control, drug-free spacer (*n* = 12)	8	8
							Colistin-loaded spacer (Coli-Ce) (*n* = 13)	8	
							Drug-free spacer + colistin intramuscular (i.m.) (Coli S) (*n* = 11)	9	
							Coli-Ce + Coli S (*n* = 12)	25	
							Coli S + meropenem subcutaneous (s.c.) (Mero S) (*n* = 12)	0	
							Coli-Ce + Coli S + Mero S (*n* = 12)	0	
[82]	Tibial component, antibiotic-loaded spacer	ns	No	Tibia (partial knee replacement, tibial component)	Prosthesis replacement by a cement spacer with or without teicoplanin, and with or without systemic antibiotic treatment, or injections of teicoplanin.	56	Untreated controls (*n* = 11)		ns
							Impl. replacement by drug-free cement spacer (*n* = 10)		
							Impl. replacement by teicoplanin-loaded cement spacer (1.2 g of teicoplanin/40 g of cement) (*n* = 12)		
							i.m. injections of teicoplanin (20 mg/kg of body weight, twice a day for 7 days) (*n* = 11)		
							Systemic antibiotic treatment combined with teicoplanin-loaded spacers (*n* = 12)		
[83]	ns	S.S.	No	Tibia (proximal metaphysis)	First revision 1 week after inoculation: group R received a spacer containing GEN and RIF microcapsules, group C received a spacer containing GEN.	15	Group C (*n* = 7)	ns	7
							Group R (*n* = 8)	ns	
[84]	Length 25 mm, diameter 3.9 mm	Preformed cement on a central metal wire	No	Tibia (medullary canal)	One-stage revision: medullary canal was debrided and washed, after which tobramycin-containing bone cement was inserted.	30	Tobramycin-containing bone cement (*n* = 10)	10	10
							Plain Simplex-P bone cement, no antibiotics (*n* = 10)	20	
							Plain Simplex-P bone cement, with systemic antibiotics (cefazolin) injected every 8 h from day 28 to 42 (*n* = 10)	0	
[85]	Kirschner wire, length 1 cm	S.S.	No	Radius (medullary canal)	Debridement with higher-dose G-PND (3.14 wt%).	16	Debridement, new wire with higher-dose G-PND (3.14 wt%) (*n* = 8)		ns
							Debridement, new wire without hydrogel (*n* = 8)		
[86]	Length 30 mm, diameter 3 mm	Metal	No	Femur (upper 1/3rd)	Two-stage revision with CWU on ALBC.	16	Revision with CWU on ALBC (*n* = 8)		ns
							Control group with ALBC but without insonation (*n* = 8)		
**Prevention: antibiotics only**									
[87]	Diameter 5 mm, length 10 mm	Tantalum	No	Radius (midshaft periosteum, with cortical damage)	Antibiotic-impregnated microspheres.	14	Infection + control limb in each rabbit (*n* = 14)		27
[88]	Diameter 2 mm, length 35 mm	Ti	No	Tibia (medullary canal)	Teicoplanin and Clindamycin coating.	30	Teicoplanin coating (*n* = 10)		ns
							Clindamycin coating (*n* = 10)		
							Uncoated coating (*n* = 10)		
[89]	Length 15 mm, diameter 2.8 mm	Ti-alloy pin	No	Femur (medullary canal)	Minocycline coating, RIF coating.	28	Minocycline and RIF coated (*n* = 14)	7	11
							Uncoated (*n* = 14)	14	
[90]	Arthroplasty implant of the first metatarsophalangeal joint (Silastic, great toe implant HP; Swanson Design, Dow-Cornin). Nail length 14 mm, implant head diameter 15 mm, height 5 mm	Silicone elastomer	No	Tibia (nail in medullary canal, head replaced tibial plateau)	At 7 days postinfection, rabbits began treatment with CPT-F (60 mg/kg of body weight i.m. b.i.d.) or VAN (60 mg/kg i.m. b.i.d.) alone or combined with RIF (10 mg/kg i.m. b.i.d.).	66	No treatment control (*n* = 14)		ns
							CPT-F (*n* = 12)		
							VAN (*n* = 12)		
							CPT-F plus RIF (*n* = 14)		
							VAN plus RIF (*n* = 14)		
[91]	Diameter 3 mm, length 40 mm, surface roughness of 7 μm	Sandblasted Ti	No	Femur (medullary cavity, intercondylar region of right femur)	DAC^®^hydrogel loaded with 0%, 2%, or 5% (*w*/*v*) VAN.	40	Histocompatibility study (*n* = 10)		ns
							High load (10^6^ CFU), 0 % VAN-loaded DAC (*n* = 5)		
							High load (10^6^ CFU), 2%VAN-loaded DAC (*n* = 5)		
							High load (10^6^ CFU), 5% VAN-loaded DAC (*n* = 5)		
							Low load (10^4^ CFU), 0 % VAN-loaded DAC (*n* = 5)		
							Low load (10^4^ CFU), 2 % VAN-loaded DAC (*n* = 5)		
							Low load (10^4^ CFU), 5% VAN-loaded DAC (*n* = 5)		
[92]	Diameter 4.1 mm, length 5 mm	Ti, coated with pure Ti powder at 0.35 mm thickness (Plasmapore)	No	Femur (in cancellous bone via lateral femoral condyle)	VAN treatment at 25 mg/kg subcutaneous neck soft tissue, twice daily for ten days.	18	MRSA, no treatment (*n* = 6)	17	11
							MRSA + treatment with VAN (*n* = 6)	17	
							MRSA, no treatment (*n* = 6)	0	
[93]	Area 20 × 5 mm^2^, thickness 0.1 mm	Ti foils	Sutured to the bone	Tibia (2 mm hole drilled in external tibial epicondyle)	VAN encapsulated in a poly(ethylene glycol) (PEG)-based hydrogel film that was covalently bound to Ti implants and subsequently covered by a PEG-poly(lactic-co-caprolactone) (PEG-PLC) membrane. Additionally, crosslinked starch (CSt) was mixed with the hydrogel.	36	2 mg VAN (*n* = 12)		ns
							4 mg VAN (*n* = 12)		
							No VAN (*n* = 12)		
[94]	Ti 0.25 × 0.25 cm, NTATi length of 1.05 µm, an inner diameter of 125 nm, and an outside diameter of 170 nm	Pure Ti and nanotubular anodized Ti uncoated (NTATi)	No	Tibia (proximal medullary cavity)	NTATi with GEN (NTATi-G), Ti coated with GEN (Ti-G), NTATi, Ti.	36	NTATi with GEN (NTATi-G) (*n* = 8)	0	11 *^1^
							Ti coated with GEN (Ti-G) (*n* = 8)	0	
							NTATi (*n* = 8)	0	
							Ti (*n* = 8)	25	
[95]	Arthroplasty implant of the first metatarsophalangeal joint (Silastic HP great toe implant; Swanson Design, Dow-Corning)	Silicone elastomer	No	Tibia (tibial plateau and metaphysis)	Levofloxacin and/or RIF from day 7 to day 14.	45	Untreated control (*n* = 10)		0
							Levofloxacin alone (*n* = 12)		
							RIF alone (*n* = 11)		
							Levofloxacin and RIF (*n* = 12)		
[96]	A tibial component (Silastic great toe implant HP; Swanson Design; Dow-Corning France, S.A.)	ns	No	Tibia (tibial plateau and medullary cavity of metaphysis)	Intramuscular injections of Q-D or VAN, with or without RIF, from days 4 to 11.	52	Q-D (*n* = 12)		ns
							Q-D + RIF (*n* = 10)		
							VAN (*n* = 10)		
							VAN + RIF (*n* = 11)		
							Untreated control (*n* = 9)		
[97]	Arthroplasty implant of the first metatarsophalangeal joint (Silastic HP great toe implant; Swanson Design, Dow-Corning) used as tibial component, stem 14 mm	Silicone elastomer	No	Tibia (tibial plateau and medullary cavity of metaphysis)	Starting 7 days postinfection, rabbits were treated with daptomycin (22 mg/kg of body weight i.v. o.d.) or VAN (60 mg/kg i.m. twice daily [b.i.d.]), alone or combined with RIF (10 mg/kg i.m. b.i.d.).	60	Untreated group (*n* = 12)	25	12
							Daptomycin (*n* = 12)	0	
							Vancoymycin (*n* = 12)	33	
							Daptomycin + RIF (*n* = 12)	0	
							VAN + RIFg (*n* = 12)	0	
[98]	Diameter 4.1 mm, length 5 mm	Ti alloy, coated with pure Ti powder at 0.35 mm thickness (plasmapore)	Polyethylene cap	Femur (through lateral condyle medullary canal)	Antibiotics received twice daily for 10 days. Linezolid orally, VAN subcutaneous injection.	36	Uninfected, no antibiotics (*n* = 6)	17	6
							Uninfected, VAN (*n* = 6)	17	
							Uninfected, Linezolid (*n* = 6)	0	
							Infected, no antibiotics (*n* = 6)	0	
							Infected, VAN (*n* = 6)	0	
							Infected, Linezolid (*n* = 6)	0	
**Prevention: surface modification**									
[99]	Diameter 3 mm, length 20 mm	Kirschner wires, Ti–6Al–4V	No	Femur (intertrochanteric crest)	Bottle-shaped TiO_2_ nanotubes (bNT).	20	Chemically polished without infection (*n* = 5)		ns
							Chemically polished with infection (*n* = 5)		
							bNT without infection (*n* = 5)		
							bNT with infection (*n* = 5)		
[100]	Length 80 mm, diameter 2.5 mm, inner drill hole 2.0 mm in slotted and 1.6 mm in cannulated nail. Slotted nail had additionally a posterior longitudinal slit of 0.4 mm	Ti–niobium–aluminum alloy (TiA16Nb7)	None	Tibia (medullary cavity)	Cannulated (CN) vs. solid (SN) and hollow slotted nail (HN).	69	Solid (SN) implant: inoculum of 4 × 10^4^ CFU (*n* = 1), 2 × 10^5^ CFU (*n* = 4), 3 × 10^5^ CFU (*n* = 6), 4 × 10^5^ CFU (*n* = 8), 2 × 10^6^ CFU (*n* = 2), 4 × 10^6^ CFU (*n* = 1)	ns	6 *^1^
							Hollow slotted (HS) implant: inoculum of 4 × 10^4^ CFU (*n* = 1), 2 × 10^5^ CFU (*n* = 4), 3 × 10^5^ CFU (*n* = 6), 4 × 10^5^ CFU (*n* = 6), 2 × 10^6^ CFU (*n* = 2), 4 × 10^6^ CFU (*n* = 1)	ns	
							Cannulated (CN) implant: inoculum of 4 × 10^4^ CFU (*n* = 1), 2 × 10^5^ CFU (*n* = 4), 3 × 10^5^ CFU (*n* = 6), 4 × 10^5^ CFU (*n* = 9), 2 × 10^6^ CFU (*n* = 2), 4 × 10^6^ CFU (*n* = 1)	ns	
[101]	Diameter 2.5 mm, length 85 mm	Ti–aluminum–niobium (TAN) or electropolished S.S. (EPSS)	No	Tibia (medullary canal, cranial to the joint)	Polished vs. non-polished nail.	72	EPSS (*n* = 19: *n* = 4 for 10^1^ CFU, *n* = 8 for 10^2^ CFU, *n* = 6 for 10^3^ CFU, *n* = 1 for 10^4^ CFU)	ns	18
							Standard TAN (*n* = 20: *n* = 4 for 10^1^ CFU, *n* = 8 for 10^2^ CFU, *n* = 6 for 10^3^ CFU, *n* = 2 for 10^4^ CFU)	Ns	
							Polished TAN (*n* = 20: *n* = 4 for 10^1^ CFU, *n* = 7 for 10^2^ CFU, *n* = 7 for 10^3^ CFU, *n* = 2 for 10^4^ CFU)	Ns	
[102]	Diameter 3 mm, length 13 mm	Grade 2 biomedical Ti	No	Femur (distal epiphysis)	Acid-etched Ti, anodic spark deposition nanostructured Ti surface, anodic spark deposition nanostructured Ti surface doped with gallium. A total of 24 implants (8 per surface) were inserted into the left and right femoral epiphysis.	12	1 week, inoculated (*n* = 2 per modification)		ns
							1 week, not inoculated (*n* = 2 per modification)		
							2 weeks, inoculated (*n* = 2 per modification)		
							2 weeks, not inoculated (*n* = 2 per modification)		
**Prevention: coating**									
[103]	Knee implant with tibial (on a screw of 20 mm long and 3.5 mm diameter) and femoral component (on a screw 15 mm long, 3–2 mm diameter)	Ti4Al6V	Screws locked	Tibia and femur (total knee implant)	Sol-gel silver coated Ti (right knee as control, left knee experimental).	26	Pilot studies (*n* = 2)	0	19
							Experimental rabbits (*n* = 24)	21	
[104]	Diameter 4 mm, length 25 mm, roughness 5.6 µm	Sand-blasted Ti	No	Tibia (medullary canal)	The rods were coated with unloaded hydrogel (Gel), hydrogel loaded with 2 % (Van2) or 5 % VAN (Van5), bioactive glass (BAG) or *n*-acetyl-L-cysteine (NAC).	42	Gel (*n* = 12)	8	5
							Van2 (*n* = 6)	0	
							Van5 (*n* = 6)	0	
							BAG (*n* = 6)	17	
							NAC (*n* = 6)	0	
							No gel (*n* = 6)	0	
[105]	Diameter 2.0 mm, length 150 mm	Ti K-wires	Advanced Surface^®^ ceramic multilayer coating	Tibia (medullary channel)	Silver multilayer coating (SML).	27	SML coated (*n* = 12)		ns
							Uncoated (*n* = 12)		
							SML coated without microbial load (*n* = 3)		
[106]	Length 25 mm, diameter 2.5 mm, lower ends bent to mimic knee prostheses	Ti alloy (Ti6Al4V)	No	Femur (medullary canal)	Implants were uncoated, hydroxyapatite-coated, or silver-coated.	27	Uncoated (*n* = 9)	22	15 *^1^
							Hydroxyapatite-coated (*n* = 9)	11	
							Silver-coated (*n* = 9)	11	
[107]	Length 25 mm, diameter 2 mm	Ti-alloy (Ti6Al4V)	No	Femur (medullary canal)	Hydroxyapatite coated or silver doped hydroxyapatite-coated implant.	33	Uncoated (*n* = 11)	18	6
							Hydroxyapatite coated (*n* = 11)	0	
							Silver doped hydroxyapatite coated (*n* = 11)	0	
[108]	Length 55 mm, diameter 3 mm	Medical grade Ti-6% aluminum-7% niobium TAN	No	Humerus (medullary cavity, entry point between greater tuberosity and deltoid ridge)	A PLEX coating containing polylactic-co-glycolic acid (PLGA); dipalmitoyl phosphatidyl choline (DPPC) and distearoyl phosphatidyl choline (DSPC); and cholesterol with doxycycline hyclate was used.	28	DoxyS + uncoated implant (*n* = 6)	17	8
							DoxyS + coated implant (*n* = 6)	0	
							DoxyR + uncoated implant (*n* = 6)	0	
							DoxyR + coated implant (*n* = 6)	17	
[109]	Total diameter 4.5 mm, length 12 mm, coating 1.5 mm thick	Solid Ti6Al4V core, coated with Ti foam	No	Tibia (proximal medullary canal)	Implants were uncoated (Ti), PeriApatite-coated, or Tobramycin–PeriApatite-coated (PA-tobra). Prior to insertion, the implant bed was contaminated with none, 10^3^, 10^4^, or 10^5^ CFU.	72	Ti (*n* = 24, ns how many per inoculum size)	0	1.4
							PA (*n* = 24, ns how many per inoculum size)	17	
							PA-tobra (*n* = 24, ns how many per inoculum size)	0	
[110]	Diameter 2.5 cm, height 0.4 cm, surface area 4.9 cm^2^	Ti-6Al-4V, grit-blasted with alumina grit	None	Femur (medullary canal, from the piriformis fossa)	HA-coated and PLGA-GEN-HA-coated pins.	14	HA-coated pins, sacrificed day 2 (*n* = 3)		ns
							PLGA-GEN-HA-coated pins, sacrificed day 2 (*n* = 3)		
							HA-coated pins, sacrificed day 7 (*n* = 4)		
							PLGA-GEN-HA-coated pins, sacrificed day 7 (*n* = 4)		
[111]	Diameter 3.9 mm, length 20 mm	Grit-blasted Ti alloy (Ti6Al4V)	No	Tibia (anterior to the insertion of the ACL, medullary canal)	Two cylinders of the same type were put in the left and right tibia. of one rabbit. Either HA-coated cylinders or uncoated cylinders (Ti).	32	HA-coated cylinders (*n* = 4 per inoculum size)	ns	6 *^1^
							Uncoated cylinders (Ti) (*n* = 4 per inoculum size)	ns	
[112]	Diameter 2 mm, length 50 mm	Ti6Al4V alloy	No	Tibia (into the medullary cavity)	HA-coated Ti6Al4V alloy with VAN-chitosan composite coating.	6	VAN-chitosan/HA composite coated (*n* = 3)		ns
							Uncoated (*n* = 3)		
[113]	Length 3 mm, diameter 2.5 mm	Ti	No	Femur (at the lower end of femur and lateral knee joint)	Both hind legs were used for implantation with Ti rods with TNT, Ag/TNT, or S-Ag/TNT structured surfaces.	15	Ti rods of TNT structured surfaces (TNT group, *n* = 10)		0
							Ti rods of Ag/TNT structured surfaces (Ag/TNT group, *n* = 10)		
							Ti rods of S-Ag/TNT structured surfaces (S-Ag/TNT group, *n* = 10)		
[114]	Diameter 2 mm, length 90 mm	Ti6Al4V Kirschner wires	No	Tibia (medullary canal 0.5 cm below the right tibial plateau and advanced to the distal end)	K-wires were coated with (1) PDLLA coating with no CuCl_2_ and (2) PDLLA coating with CuCl_2_.	24	PDLLA coating, no Cu, with saline (*n* = 6)		4
							PDLLA coating, with 1.0 mg/mL CuCl_2_, with saline (*n* = 6)		
							PDLLA coating, no Cu, with bacteria (*n* = 6)	17	
							PDLLA coating, with 1.0 mg/mL CuCl_2_ with bacteria (*n* = 6)		
[115]	Diameter 3 mm, length 5 mm	Ti medical grade	No	Femur (transverse defect at distal side)	ZnO nanorods are first synthesized on the Ti substrate and then partially converted into Zn_3_(PO_4_)_2_.	ns	Ti (*n* = ns)		ns
							Ti-ZnO (*n* = ns)		
							Ti-ZnP2 (*n* = ns)		
[116]	Diameter 2 mm, length 10 mm	Ti	No	Femur (medullary cavity)	MNRs on Ti were developed, with fixed interrod spacing of about 70 nm, and fluorine (F) incorporated Ca_9_Sr_1_(PO_4_)_6_(OH)_2_ (Sr_1_−HA, strontium containing hydroxyapatite) with the fixed Sr but different F content.	24	Ti (*n* = 4)		ns
							MNR-F0 (*n* = 4)		
							MNR-F1 (*n* = 4)		
							MNR-F2 (*n* = 4)		
							MNR-F5 (*n* = 4)		
							MNR-F7 (*n* = 4)		
							Ti + PBS (*n* = 4)		
[117]	Diameter 2 mm, length 6 mm	Ti	No	Femur (two holes (φ 2 mm) were drilled on each leg)	Implant coated with polyethylene glycol (PEG)-based thermoresponsive polymer (MPEG) and an antimicrobial peptide (AMP) HHC36.	ns	Ti (*n* = ns)		ns
							Ti-M2 (Ti treated with MPEG2 solution) (*n* = ns)		
							Ti-A (Ti treated with HHC36 peptide solution) (*n* = ns)		
							Ti-M2-A (Ti-M2 treated with HHC36 peptide solution) (*n* = ns)		
**Other**									
[118]	Screw 4 × 14 mm, with U-shaped washer	S.S. screw, UHMWPE washer	No	Femur (lateral femoral condyle)	Seven days after inoculation each knee was lavaged twice. Each rabbit had one experimental knee with 3.5% dilute Betadine solution, and one control knee with normal saline.	8	*n* = 8	0	0
[119]	Kirshner wire, length 1 cm	Stainless steel	No	Radius (1 cm defect was created)	PNDJ1.51 with tobramycin, low-dose antimicrobial loaded bone cement (ALBC) with tobramycin, systemic tobramycin.	30	PNDJ1.51 with tobramycin (*n* = 6 MSSSA)	ns	13
							PNDJ1.51 with tobramycin (*n* = 6 MRSA)	ns	
							Low-dose antimicrobial-loaded bone cement (ALBC) with tobramycin (*n* = 7 MSSA)	ns	
							Systemic tobramycin (*n* = 7 MSSA)	ns	
[120]	Length 40 mm, diameter 3.5 mm	Ti	No	Tibia (proximal metaphysis and diaphysis)	Smooth Ti, grit-blasted Ti, HA-coated Ti, trabecular metal, cancellous Ti rods.	50	Smooth Ti (*n* = 10)		0
							Grit-blasted Ti (*n* = 10)		
							HA-coated Ti (*n* = 10)		
							Trabecular metal (*n* = 10)		
							Cancellous Ti rods (*n* = 10)		
[121]	Ab-PLGA screw: outer diameter 2.7 mm, length 24 mm, pitch 0.0 mm. (Group I and III). Control screw: diameter 2.7 mm, length 14 mm, pitch 1.0 mm (Group II and IV)	Ab-PLGA: self-reinforced ciprofloxacin containing poly(lactide-co-glycolide) 80:20. Control: standard S.S.	Screwed into bone	Tibia (proximal metaphysis)	Ab-PLGA screw with ciprofloxacin or a S.S. screw. The surgical field was lavaged with 100 mL of sterile saline in the inoculated groups. In negative control animals (no inoculum), a similar lavage of the wound space was performed, but with 150 mg of cefuroxime sodium.	24	Ab-PLGA screw + *S. aureus* (*n* = 8)		ns
							SS screw + *S. aureus* (*n* = 8)		
							Ab-PLGA (*n* = 4)		
							SS screw (*n* = 4)		
[122]	Screw and washer	s.s. screw, UHMWPE washer	Cement	Femur (tunnel was created through condyle)	Administration of AZD6389 * or IgG1, as control, 12 h before inoculation.	26	AZD6389 * (*n* = 13)		ns
							Control (*n* = 13)		
[123]	Length 2 cm, diameter 0.2 cm	Ti–CU sintered alloy, pure Ti as control	No	Femur (medullary cavity)	Ti–Cu nail (Cu–Ti/Ba) or pure Ti nail with infection sacrificed either at day 1, 7, 14, or 28.	24	Cu–Ti/Ba (*n* = 3 per day of sacrifice)		ns
							Ti/Ba (*n* = 3 per day of sacrifice)		
[124]	Screw diameter 3.5 mm, length 15 mm. Washer inner diameter 3.5 mm, external diameter 8 mm, thickness 1.5 mm	s.s. screw and UHMWPE washer	No	Femur (lateral femoral condyle)	Lavage with allicin, with or without VAN (14 days after inoculation).	32	Normal saline (*n* = 8)		ns
							VAN (*n* = 8)		
							Allicin (*n* = 8)		
							Allicin with VAN (*n* = 8)		
[125]	Silastic implant (Dow Corning, Midland, MI, USA), length 14 mm, implant head 15 × 5 mm	Silicone	No	Tibia (medullary cavity, replacing tibia plateau)	Injected with 1 mL of bacteriocin suspension or saline.	12	Bacteriocin (*n* = 6)		ns
							Control, saline (*n* = 6)		
[126]	2 mm diameter, 15 mm long	Kirschner wires	No	Femur (medullary canal, through intercondylar notch)	Bone marrow stem cells (BMSCs) infected with the recombinant PR-39 lentiviruses (pLV/PR-39).	24	BMSCs infected with pLV/PR-39 (*n* = 12)	ns	4
							Control, BMSCs infected with pLV/EGFP (*n* = 12)	ns	

*^1^ Rabbits were replaced in these studies.

### 4.3. Outcome Measures

What outcome measures are relevant for an experiment depends on the research goal. Bacterial culture and health monitoring are standard when investigating new antibacterial technologies against PJI in a rabbit. Hematology, histology, and imaging can study infection and bone growth more extensively. Table 4 and Table 5 show all outcome measures implemented by the studies included in this review and are discussed below. The range of possible outcome measures is extensive. Nevertheless, it is essential to adhere to the 3Rs principle [38]. Increasing the number of outcome measures often necessitates including additional rabbits in the experimental groups. Therefore, the scientific value gained from including additional outcome measures must justify the increased burden on the animals or a larger group size.

#### 4.3.1. Bacterial Culture

In a PJI rabbit model, bacterial culture is the most important outcome measure when studying bacterial adhesion and biofilm formation and its prevention. Bacterial culture can confirm or negate and quantify an infection and assess the antimicrobial efficacy of the novel antibacterial compound. Moreover, culturing is still the gold standard in diagnosing PJI in the clinic, and implant sonication is the most likely diagnostic test to confirm PJI [151]. Remarkably, three studies included in this review did not report on any bacterial culture. A significant amount of variation between the cultured tissue, the culturing method, and the unit of measurement was observed among the studies included in this review.

The cultured tissue in the studies included in this review ranges from one swab to culturing multiple tissues. The most cultured tissues are the implant, bone tissue, soft tissue, synovial fluid, and periprosthetic tissue. A blood sample may also be cultured to detect systemic infection. The samples used for the culture depend on the working mechanisms of the antibacterial technology and the study’s objective. One article included in this review did not specify what material they cultured [119]. The cultured tissues vary the most for the studies included in this review that did not use an intervention against PJI. Craig et al. [69] cultured samples from the arterial blood; joint capsule; synovial scar surrounding the screw, surrounding bone, liver, and kidney samples; and the UHMPHE washer, screw, and affixed bone cement complex. However, this was their only outcome measure other than basic health monitoring. Other studies only cultured the exudate around the implant (Sarda et al. [74], Sarda-Mantel et al. [75], Tang et al. [76]); however, they tried to visualize the infection using 99mTc scintigraphy and used the culture as a control. This demonstrates that the selection and number of cultures to perform depend on the study’s objective and the other employed outcome measures. For the studies included in this review that used revision, antibiotics, surface modifications, coatings, or other antibacterial techniques, the antibacterial working mechanism is leading for the cultures. Brunotte et al. [79] studied a spacer during two-stage revision; therefore, they cultured both implants and the spacer to track bacterial growth over time. For antibacterial technologies that depend on surface modifications or a contact-killing or anti-fouling coating, the most important outcome is if bacteria are still growing on the implant. However, it is still interesting if bacteria survive in the tissue around the implant, even though they cannot survive on the implant itself. Interpretation of this data might be difficult, as a negative culture of the implant, combined with a positive culture of tissue surrounding the implant, does not mean the bacterial technology does not sufficiently work. All studies included in this review that researched surface modifications cultured the implant and the bone or interfacial tissue surrounding the implant. For coatings or techniques that leach the antibacterial compound into the tissue surrounding the implant, both the implant and surrounding tissue should be cultured. Unfortunately, the rabbits used for tissue culturing cannot be used for histology or imaging for which the implant must be taken out or slices of the limb must be made. Therefore, it is inefficient and wasteful to use a rabbit for a single tissue culture when multiple cultures could provide more information. The suffering of the animals should yield as much quantifiable data as possible. Conducting multiple cultures can aid in quantifying the bacteria and determining their distribution.

The culturing method, and the unit of measurement this results in, vary greatly too. Several studies use a swab method, from which it can be concluded if a site was infected or not. Overstreet et al. [85] and Zhang et al. [114] used swabs as their sole bacterial culture method. However, relying solely on swabs can overlook deeper-seated bacteria, result in false negatives when bacterial counts are low, and the results are dependent on the technique and the specific location swabbed. Most studies included in this review that used a swab also plated out samples, generating quantifiable data. In these instances, swabs can offer additional information on less critical sites. Plating out the sonicate of the implant or homogenized soft tissue or bone is commonly performed. The culturing of these fluids differs per study and laboratory. However, the plating of serial dilutions is the most common and results in the exact CFU per sample. There are exceptions, like Brunotte et al. [79], who rolled the implants over an agar plate, or Yu et al. [123], who counted until a maximum of 1000 CFU. Serial dilution of samples should be performed in all studies, as it provides clear, quantifiable data and aids in distinguishing between experimental groups. Of the studies included in this review, 10% did not report the used culturing method [80,87,89,98,104,119], and 10% did not report the unit of their outcome measure [73,81,83,85,90,127].

#### 4.3.2. Health Monitoring

Health monitoring, including measuring the weight and temperature of the rabbits and checking for clinical signs of infection, is important for maintaining and assessing animal welfare (refinement) [64]. Animal welfare monitoring is important in deciding whether a humane endpoint has been met [45]. Among the studies included in this review, 28% did not report any health monitoring. Of the 60 studies, only 26 reported the rabbits’ weights, 25 reported the rabbits’ temperatures, and 28 reported clinical signs of infection. Whereas temperature and weight are standard physiological markers of illness, there is a wide range of clinical signs of infection that researchers can look for, as presented in Table 4 and Table 5. As stated by Mapara et al. [33], health monitoring should include assessing if the rabbit is bright, alert, active, inquisitive, has a smooth coat, and a good body condition. Pain or infection may be shown as a change in gait, abnormal weight distribution, retraction of injuries, changed postures, swelling, inflammation, decreased activity, bad wound healing, or decreased food and water intake. Exorbitant pain may lead to shock or abstinence from eating, which may lead to death [33]. Monitoring the rabbits’ health may lead to preventative care to maintain the welfare of the rabbits, like administering extra analgesia or force-feeding. A well-designed score sheet may help the caretakers of the rabbits to assess their health objectively and should be used in all animal studies [24,152]. Multiple score sheets already exist, like the Bristol Rabbit Pain Scale [153] and the Rabbit Grimace Scale [154]. The ARRIVE guidelines also state that welfare-related assessments and health status should be documented [43,44].

**Table 4:** Outcome measures extraction results—part 1. Abbreviations used: ns = not stated.

Reference	Bacterial Culture			Health Monitoring (1 = Measured Daily, 2 = Measured Weekly, 3 = Post-Mortem, 4 = Not Stated When)			Hematology (1 = Measured Daily, 2 = Measured Weekly)			
	Tissue Cultured	CULTURE Method (1= Homogenized (For Bone) or Vortexed (For Implant), Sonicated, Serial Diluted, and Plated)	Outcome Unit	Weight	Temperature	Clinical Signs of Infection a. Swelling b. Inflammation c. Activity d. Food Intake e. Wound Healing f. Pus g. Clinical Signs of Infection	C-reactive Protein (CRP)	Erythrocyte Sedimentation Rate (ESR)	Leucocyte WBC/Differentiation	Interleukin-6 (IL-6)
**No intervention used**										
[69]	Arterial blood, joint capsule, synovial scar surrounding the screw, UHMPHE washer, screw with bone cement complex, surrounding bone, liver and kidney samples	Biopsies, sonicated and plated	CFU/g of tissue, CFU/mL joint fluid, CFU/unit for the screw–washer complex		1	Inside joint and fluid were graded on a three-point scale of infection				
[70]	Tibial plateau at point of insertion and nail and bone surrounding the nail	Swabs and sonication	CFU	2	2	4, c, d, g			2	
[71]	Intra-articular samples, and sample from bone (tibial canal), soft tissue (synovial and capsule), and implant	Sonicated, seeded on agar plates	CFU	At time of inoculation and 7 days thereafter	At time of inoculation and 7 days thereafter	4, f, macroscopic appearance of the joint, fistula, or other wound complications. Knee bending and weight-bearing		Yes	Yes	
[29]	Knee joint cavity and tibial plateau and tuberositas tibiae	Swabs and tissue removal, homogenized, cultured		2, and on the day of surgery	2, and on the day of surgery	4, b, e, a, use of hindlegs	Yes	Yes	Yes	
[72]	Bone	Cultured	Bacterial growth yes/no	4		4, weight bearing on the operated leg	Yes	Yes		
[73]	Bone with marrow	Homogenized		Before implantation, inoculation, and sacrifice	Before implantation, inoculation, and sacrifice	1, c, d, e, g		Before implantation, inoculation, and sacrifice	Before implantation, inoculation, and sacrifice	
[74]	Exudate around prosthesis	Spread onto blood-agar	Infection yes/no			3 arthritis, osteitis, and tibial myelitis analysis				
[75]	Exudate around prosthesis	Spread onto blood-agar	Infection yes/no							
[76]	Exudate around prosthesis	Cultivated on blood agar for 72 h	CFU yes/no			3, b, f, joint effusion, abscess formation, cortical lysis				
[77]	Implant and knee joint	1	log10 CFU/joint or implant	2	2		2			
[78]	Soft tissue	72 h growth	Bacterial growth yes/no				Biweekly			Biweekly
[30]	Implant	Sonicated and cultured	CFU	Every 48 h	Every 48 h		Day 3/5/7/14/21/28	Day 3/5/7/14/21/28	Day 3/5/7/14/21/28	
**Revision**										
[79]	Removed K-wires (day 28 and day 84) and spacers (day 56)	Rolled on agar plate and sonication with plating	CFU/mL	1		a				
[80]	Implant		Positive yes/no			4, c, d, g, local pain. formation of fistulae				
[81]	Upper third of tibia	Crushed, pulverized				Skin aspect was noted 14 days after inoculation				
[82]	Bone	Homogenized, plated	CFU/g of bone							
[83]	Intra-articular culture and Bone, soft tissue, and spacers	ns and sonicated		Day 0, 8, 11, 15, 22, 29, 36	Day 0, 8, 11, 15, 22, 29, 36	Checked for fistulas in contact with articulation		Day 0, 8, 11, 22, 29, 36	Day 0, 8, 11, 22, 29, 36	
[84]	Bone	1	CFU/g of bone	Regularly	Regularly			2	2, WBC	
[85]	Wire and biopsy of adjacent tissue	Swab cultures				a				
[86]	Cortical bone, bone marrow, muscle tissue, bone, and synovial fluid	Homogenized and cultured	Scored 0–10	Day 1, 30, 60	Day 1, 30, 60	3, g, pain on palpation, abscess	Day 1, 30, 60	Day 1, 30, 60		
**Prevention: antibiotics only**										
[87]	Tissue and pus or hematoma present adjacent to the implant		Positive of negative culture	Before euthanization	1	4, a, redness, ambulatory status, favoring limb				
[88]	Swabs of entrance of implant and intramedullary canal. Bone, implant	Swabs cultured. Bone: 1	Bacterial growth yes/no	1						
[89]	The implanted femoral bone swabs, blood sample	Swabs	CFU			4, e, g, mobility, ability to thrive				
[90]	Prosthesis and bone	Smear and crushed, pulverized								
[91]	Intra-medullary femur, blood sample	Swab, culture	CFU/mL							
[92]				Day 0, 7, 14, 21, 42	Day 0, 7, 14, 21, 42				Pre-op and 6 weeks post-op	
[93]					2, minus week 3				WBC, 1, 2, 4 weeks	
[94]	Tibia and bone tissue	Rolled in agar and cultured in broth	CFU on agar/cloudiness of broth	Days 0, 3, 7, 14, 21, 28, 35, 42	Day 0, 3, 7, 14, 21, 28, 35, 42	4, clinical signs of infection				
[95]	Tibia	1	CFU/g bone							
[96]	Upper 1/3rd of Tibia	1	CFU/g of bone							
[97]	Implant, Tibia	Implant smear. Bone: 1	Sterile yes/no, log10 CFU/g of bone							
[98]	Bone and tissue		Infection yes/no							
**Prevention: surface modification**										
[99]	Implant and bone	Sonication, plated	CFU/g bone, CFU/cm^2^ implant	1	1	Pain and stress of the rabbits were observed				
[100]	Bone and implant	Bone crushed and spread on agar plate; implant rolled on agar plate	Qualitative	1	1					
[101]	Nail and proximal tibial bone that surrounded the nail	Vortexed, sonicated, plated	CFU/nail or CFU/bone fragment	First 3 days, and on days of blood sampling	First 3 days, and on days of blood sampling				Day 0 and 72, weekly until the end of the study	
[102]	Implant surface and interfacial tissue exposed along the implant	Swabs and tissue samples plated	CFU							
**Prevention: coating**										
[103]	Implant + swab and irrigation from knee	Cultured	Infection yes/no	2	2	4, general well-being, posture				
[104]	Anterior bone fragments		CFU/g of bone					Week 1, 2, 3, and 4 post-op		
[105]	Implant and bone marrow	Sonicated/vortexed and plated	CFU/g of bone/marrow			3, a, b, f, edema, bone marrow quality				
[106]	Medullary canal, implant	Swab from canal, implant: 1	Positive culture yes/no, CFU/mL		2	1, c, d, e				
[107]	Intramedullary canal and rods	Swab culture and sonication and plating	CFU/cm^2^			4, c, d, e	Before surgery, week 2, 6, 10			
[108]	Implant and humerus	1	CFU/bone fragment or implant	2, and day 3			2, and day 3		2, and day 3, WBC	
[109]	Tibia	1	CFU/g of bone	1	1	1, c, d, e		2	2	
[110]	Bone, implant	1	CFU/g of bone, CFU/implant	4	4	1, g, abscess formation, cortical lysis			Midway, end of study, WBC and differentiation	
[111]	Tibia	1	CFU/g of bone	4	4	4, c, d, e		Yes	WBC	
[112]	Blood, knee joint, and tibia marrow	Cultivated on blood agar	Bacterial growth yes/no							
[113]	Implant	Sonicated and plated	Bacterial growth yes/no							
[114]	Implant	Swab culture	Qualitative assessment		Regularly		2		2, WBC	
[115]	Implant	Sonicated and plated, and turbidity measured	CFU							
[116]	Implant and femur	1	CFU							
[117]	Implant and marrow	4 h culture in broth then plated	CFU							
**Other**										
[118]	Blood, implant, bone, joint capsule	1	CFU/g of biopsied tissue	1		1, e, distress				
[119]			Sterile yes/no			4, a, f				
[120]	Implant, bone–implant interface, metaphyseal bone	Conventional cultures and PCR	CFU			1, d, e, g, physical condition				
[121]	Subfascial soft tissues, screw heads, screw tract, and removed screws	Swab cultures and incubation in broth	CFU/g of bone	Yes, in week 3 and 6		4, a, and erythema				
[122]	Implant, joint capsule, all infected synovial tissue	Sonicated and cultured	log10 CFU			3, a, f, erythema,				
[123]	Implant and surrounding tissue swabs	Cultured in medium	CFU, max of 1000		1	1, incision redness, swollen, exudate		Day 1/4/7/14	WBC day 1/4/7/14	Day 1/4/7/14
[124]	Screws	Washed, sonicated, plated	CFU/mL	4	4					
[125]							1			1
[126]	Bone	1	CFU				Day 1, 3, 7, 14	Day 1, 3, 7, 14		

**Table 5:** Outcome measures extraction results—part 2.

Reference	Histology (Stained Area Specified)				Imaging				Other
	H&E Staining	Other	Infection Scoring (1 = Qualitative, 2 = Semi-Quantitative Ordinal scoring)	Bone Apposition Scoring	X-ray		Other		
					Pre-Mortem	Post-Mortem	Pre-Mortem	Post-Mortem	
**No intervention used**									
[69]									
[70]	Yes	Brown and Brenn stain	1		Post-operative to assess placement of implant	To assess migration of implant and signs of osteomyelitis			
[71]									
[29]		Masson–Goldner or Gram staining	2		After 6 weeks, osteomyelitis scoring system			µCT with osteomyelitis scoring system	Fluorescence microscopy, 3 different calcium-binding fluorophores were administered at week 2 (calcein green), 4 (xylenol orange), and the day before sacrifice (calcein blue)
[72]		Masson–Goldner or Gram			Weekly, periosteal elevation, cortical thickening, and osteolysis		18F-FDG uptake PET before and at week 1, 3, and 6, infection	µCT, ex vivo, infection yes/no	
[73]		Gram staining	2			Yes			PCR to reveal the presence of *S. aureus* DNA. RT-PCR to confirm viability of microorganisms
[74]							99mTc-Ciprofloxacin Imaging, 5/12/19 days after surgery		
[75]							99mTc-UBI 29-41 scintigraphy day 9 and 20		
[76]	Yes		1				MRI day 7 and 21, biodistribution of 99mTc-annexin V		
[77]							Weekly 18F-FDG and 68Ga-FAPI PET/CT, SUVmax, SUVmean, MTV, and total lesion glycolysis/total lesion fibrosis		Change in knee width pre- and post-operatively
[78]	Yes	IHC for CD45 and FAP	1				Biweekly 18F-FDG and 68Ga-FAPI PET/CT [SUVmax, SUVmean, MTV]	µCT for BS/BV, BS/TV, BV/TV	Pullout strength, FAP
[30]	Yes	Modified Gram’s	1		Weekly for Friedman assessing				
**Revision**									
[79]	Yes	Toluidine-blue, Gram staining	1						
[80]	Yes		1			Yes, bone thickness, sclerosis, cysts, diaphysitis			
[81]									
[82]									
[83]									
[84]					After first operation, and before and after revision surgery on day 28, reactive bone tissue and infection				PCR for bacterial DNA in tibial cortex
[85]	Yes								
[86]	Yes	Gram	2						
**Prevention: antibiotics only**									
[87]		Methylene blue		Tissue ingrowth [%]		Yes, radiographs of both forelimbs			
[88]									
[89]									
[90]									
[91]									Toluidine blue, acid fuchsin, and fast green used for histocompatibility study only, not infection model
[92]	Yes	Masson-Goldner trichrome	2						
[93]	Yes		1		1 and 2 weeks, inflammatory response				
[94]	Yes	Toluidine-blue	2		X-rays taken on days 0, 7, 14, 21, 28, 35 and 42. Seven inflammatory criteria were scored, maximum score of 17				
[95]									
[96]									
[97]									Mutant-resistant MRSA sought in positive cultures (defined as having 3-fold-increased MICs)
[98]		Masson–Goldner trichrome		Calcified and non-calcified tissue around implant [%]					Mechanical testing of implant stability
**Prevention: surface modification**									
[99]	Yes			Osseointegration, bone–implant membrane interface, polymorphonuclear cellularity per high-power field. Complete, partial, non-existent osseointegration					
[100]		Gram	1		After operation and 28 days, for osteomyelitis				
[101]									
[102]	Yes	Masson’s trichrome and Gram staining.		Bone to implant contact (BIC) (%), Bone volume (BV) (%), Mineralizing volume (MV) (%)	Immediately after surgery to verify implant location	After sacrifice to evaluate bony tissue adjacent to implant			Fluorescence microscopy: calcein green on 5th and 6th day to all animals, xylenol orange on 12th and 13th day only to 2 week animals
**Prevention: coating**									
[103]									
[104]		Fuchsin and methylene blue	2					µCT, analysis of bone apposition on the implant surface (% of bone–implant contact)	Injections of fluorochromes (xylenol orange and calcein green) were used to visualize dynamic bone formation. At day 3 and 10 or day 7 and 21
[105]	Yes	Gram	2						
[106]	Yes		1			Osteolysis around implant scored			
[107]	Yes	Masson’s trichrome	1						
[108]	Yes	Brown–Brenn	1		Day 1 and 7, for infection signs			Head of nail used for SEM	
[109]		Fuchsine and methylene blue	2	Bone–implant contact, bone area	Check implant position				
[110]									
[111]		Basic fuchsin and methylene blue	1	Bone–implant contact, bone area [%]					
[112]	Yes		1						
[113]	Yes	Masson trichrome	1		Week 2 and 4 for infection signs		Week 1 and 2 radionuclide bone scanning for inflammation signs		
[114]	Yes		2			Yes, for fracture healing and callus index			
[115]	Yes	Giemsa staining for bacteria.						µCT [BV/TV, BS/BV, tTb.Th, Tb.*n*, and Tb.Sp] and FE-SEM of implant	Fluorochromes staining with alazarin red and calcein for bone formation and methylene blue acid magenta
[116]		Van Gieson’s picrofuchsin		Bone-to-implant contact					Pullout strength
[117]	Yes	Methylene blue and basic fuchsin		Area of fibrous connective tissue at bone–implant interface				µCT [BV/TV, tTb.Th, Tb.*n*, and Tb.Sp]	
**Other**									
[118]									
[119]									
[120]						Ex-vivo fluoroscopy for infection/osteolysis			
[121]	Yes					Sequestral bone formation, periosteal new bone formation, destruction of bone, screw loosening, peri-implant reaction, soft-tissue calcification, and swelling evaluated, numerical score was assigned for each variable	18F-FDG-PET imaging, for imaging of biomaterial-related infection		
[122]								SEM of implant	Total weight of infected synovial tissue
[123]	Yes		1		Day 1/7/14/28 for evaluation of periosteal reaction, osteolysis, or abscess formation				
[124]								SEM of washer surface biofilm formation	
[125]		0.01% acridine orange							
[126]	Yes				Day 14, to check bone density				

#### 4.3.3. Hematology

As rabbits are prey animals, they hide or show few clinical signs of illness [33,155]. Hematology might provide extra information about infection progression and the health of the rabbit, without sacrifice. The most common hematology parameters to test in PJI research are C-reactive protein (CRP), erythrocyte sedimentation rate (ESR), and white blood cell count (WBC), optionally including leukocyte differentiation [156,157]. Only 38% of the studies included in this review used hematology. Twelve studies measured CRP, thirteen measured ESR, sixteen measured WBC, and three measured IL-6.

Both CRP and ESR are non-specific markers for PJI, and could also be elevated from post-operative inflammation [156]. Several studies included in this research that compared uncontaminated and contaminated implants have also shown that, in rabbits, a rise in CRP, ESR, and WBC correlates with PJI [30,72,73,127]. As leukopenia can also be a stress response, changes in leukocyte differentiation are a better indication of infection than WBC alone, e.g., a deviation from the 1:1 ratio between neutrophils and lymphocytes [155,158]. Furthermore, monocytosis may indicate chronic inflammation, although, from a normal monocyte count, it cannot be concluded that inflammation is not present [155]. Odekerken et al. [127] compared an uncontaminated and contaminated implant in NZW rabbits, and found a lower lymphocyte count, a higher neutrophils count, and monocytosis in the contaminated group. Interleukin-6 (IL-6) is potentially a more accurate parameter; however, clinical studies that report on this marker in PJI are limited [157]. Wang et al. [78] studied PJI in NZW rabbits, and found higher IL-6 values for the contaminated group compared to the uncontaminated group. Interpretation of hematological parameters in rabbits might be difficult, as prolonged stress might influence these parameters [155]. CRP, ESR, WBC, and IL-6 are not accurate enough to conclude that an infection is present or not, but they indicate there might be an infection and should be used as supplements to other outcome measurements [159].

#### 4.3.4. Histology

Different stainings can aid in scoring areas surrounding the implant for infection or bone apposition. The choice of staining depends on the goal of the study. More than half (60%) of the studies included in this research used histology as an outcome parameter, of which Hematoxylin and Eosin (H&E) was the most popular. Due to the wide variety of staining techniques available, the study’s objective should guide the selection of the appropriate staining method. Table 6 presents an overview of all stainings used in the articles included in this review, and what tissues are colored. As preparing and analyzing the histological samples is technically difficult, the expertise available can also guide in selecting the appropriate method. Unfortunately, histology cannot be performed in the same rabbits used for bacterial culture, and longitudinal studies are not possible in the same animal, increasing the sample size. The variability and reliability of the results can be impacted by variations in staining techniques and the quality of the tissue samples. However, including histology in a study allows for a detailed examination of tissue and cellular composition, adding an understanding of the working mechanism of the antibacterial technologies compared to untreated groups. Histology can indicate the extent of infection, inflammation, and tissue damage, providing a comprehensive understanding of the pathological changes associated with PJI.

**Table 6:** Overview of various staining techniques and their application used in the articles included in this review.

Staining	Tissue Stained (Color)	References
Hematoxylin and Eosin (H&E)	Nuclei (blue) Cytoplasm and extracellular matrix (pink) Condensation of hematoxylin in nuclei is cell-specific Differentiates between osteocytes, osteoblasts, chondrocytes, and fibroblasts	[30,70,76,78,79,80,85,86,92,93,94,99,102,105,106,107,108,112,113,114,115,117,121,123,126]
(Modified) Gram	Gram-positive bacteria (purple-brown) Gram-negative bacteria (red) Eukaryotic cells do not stain	[29,30,72,73,79,86,100,102,105]
Brown Brenn	Gram-positive bacteria (blue) Gram-negative bacteria (red) Nuclei (red) Background tissue (yellow)	[70,108]
(Modified) Masson–Goldener trichrome	Collagen fibers (green/blue) Muscle fibers (red) Cytoplasm (red/pink) Nuclei (dark brown/black) Differentiates between calcified and non-calcified tissue	[29,72,92,98,102,107,113]
Fuchsin and methylene blue	Nuclei (blue) Cytoplasm (red/pink) Cartilage (blue to purple)	[87,104,109,111,117]
Toluidine-blue	Stains specific structures in tissues differently Used to visualize pathological and cortical bone formation, muscle, and bone sequesters	[79,94]
0.01% acridine orange	Visualize biofilm	[125]
Van Gieson’s picrofuchsin	Bone tissue (red) Fibrous tissue (yellow)	[116]
Giemsa	Eukaryotic cells (purple) Bacterial cells (pink)	[115]

#### 4.3.5. Imaging

Contrary to histology, for which the group size must increase, imaging is a non-invasive method to provide extra information without sacrificing the rabbits (Table 7). Furthermore, new technologies are being developed to track infection or bone growth over time. Half of the studies included in this review did not incorporate any imaging techniques. As radiography is a standard diagnostic procedure, it is the most used imaging technique in the studies included in this review. X-ray is a widely available and relatively inexpensive imaging option. Fourteen studies used X-ray pre-mortem, and nine studies used it post-mortem. X-ray is used to study placement and migration of the implant; signs of osteomyelitis; periosteal elevation; osteolysis; bone thickness; sclerosis; cysts; diaphysitis; soft tissue swelling and calcification; deformity; sequestrum formation; spontaneous fracture; callus index; and abscess formation. Several studies have used imaging techniques pre-mortem. PET scanning was used independently for 18F-fluorodeoxyglucose (18F-FDG) (Mäkinen et al. [121]), including CT and 68 Ga-fibroblast activation protein inhibitor (68Ga-FAPI) (Wang et al. [77,78]), or including µCT (Odekerken et al. [72]). 18F-FDG is a glucose analog and radioactive tracer, and it is taken up by cells with high glucose demand, such as inflammatory cells [160]. All four studies showed a higher 18F-FDG uptake in the infected groups. Both studies by Wang et al. [77,78] showed greater sensitivity to 68Ga-FAPI in detecting infection compared to 18F-FDG. Unfortunately, PET scans are significantly more expensive than X-rays for rabbits, reflecting the specialized equipment required, compared to the more routine and widely available X-ray procedures. Technetium-99m (99mTc) has been used to detect infection by labeling it to annexin V, which binds to apoptotic cells (by Tang et al. [76]); ciprofloxacin, which targets living bacteria by binding to DNA gyrase (by Sarda et al. [74]); and ubiquicidin (UBI) 29-41, an antimicrobial peptide that binds to bacterial cell membranes (by Sarda-Mantel et al. [75]). 99mTc-ciprofloxacin accumulation was found in both infected and uninfected joints in the rabbits; however, 99mTc-annexin V and UBI29-41 could differentiate between infected and uninfected joints. Both the study by Tang et al. [76] and the study by Sarda-Mantel et al. [75] state that more research is needed before 99mTc labeling can be used as a diagnostic tool. Zhang et al. [113] used 99Tc-MDP, the stable end product of the decay of 99mTc, for bone scanning and visualization of areas with increased bone turnover. Tang et al. [76] also used MRI to visualize tissue changes around the prosthesis.

In addition to X-rays, µCT and SEM were used as post-mortem imaging techniques. After excision of the extremity including the implant, µCT can be implemented for both infection and bone apposition scoring. Bone apposition on the implant surface can be measured using the bone and tissue volume, and bone histomorphometry can be analyzed using the trabecular thickness, number, and separation [78,104,115,117]. µCT can give detailed insights into the bone (micro)structure and provide quantitative data. As the extremity needs to be excised and can be fixed, µCT is widely available, as samples can be sent to different laboratories. Several studies used SEM imaging to visualize the formed biofilm on the implant [108,115,122,124]. SEM can provide good visualization of the disposition of bacteria on the implant and the formed biofilm. However, bacterial adhesion cannot be quantified. SEM imaging is performed after explanting and fixating the implant, making the implant unusable for further bacterial cultures.

**Table 7:** Overview of imaging methods used by the articles included in this review. The outcome measures, including what is exactly visualized, are specified.

Imaging Method	Use	Pre- or Post-Mortem	References
X-ray	Placement and migration of the implant; signs of osteomyelitis; periosteal elevation; osteolysis; bone thickness; sclerosis; cysts; diaphysitis; soft tissue swelling and calcification; deformity; sequestrum formation; spontaneous fracture; callus index; and abscess formation	Both	[29,30,70,72,80,84,87,93,94,100,102,106,108,109,113,114,120,121,123,126]
PET (18F-FDG)	18F-FDG is a glucose analog and radioactive tracer and is taken up by cells with high glucose demand, visualizing inflammatory cells. PET 18F-FDG can be combined with, (µ)CT or 68Ga-FAPI	Pre-mortem	[72,77,78,121]
Scintigraphy with Technetium-99m labelling	Bone scanning, detects infection when labeled to Annexin V (binds to apoptotic cells); Ciprofloxacin (targets living bacteria); Ubiquicidin (binds to bacterial cell membrane)	Pre-mortem	[74,75,76]
Scintigraphy with 99Tc-MDP labeling	Bone scanning and visualization of areas with increased bone turnover.	Pre-mortem	[113]
µCT	Infection and bone apposition scoring. Can give detailed insights into the bone (micro)structure (bone and tissue volume, and bone histomorphometry can be analyzed using the trabecular thickness/number/separation). Provides quantitative data	Both	[78,104,115,117]
SEM	Visualize formed biofilm on implant	Post-mortem	[108,115,122,124]

Choosing an imaging technique is again dependent on the research goal of a study. However, it is also dependent on the availability of the imaging apparatus. Pre-mortem imaging techniques add valuable information, and animals can be followed over time as they do not need to be sacrificed. X-rays are available at most animal research institutes and may provide valuable information at a relatively low cost. Other pre-mortem techniques might provide more information about bone growth or infection; however, they are not available at all institutes and come with higher costs. SEM is more easily available; however, it requires sacrificing the rabbits.

#### 4.3.6. Other Outcome Measures

The studies included in this review have measured several other outcomes that are less standard for a PJI rabbit model. Latex agglutination tests are commonly used to detect if *S. aureus* is present; however, they cannot differentiate between strains and sometimes give false negatives for MRSA [161]. Poultsides et al. [73] and Nijhof et al. [84] used PCR to check if the bacteria present in their bacterial cultures was the same strain as they injected at the start of the experiment or if the rabbit was infected with another strain. However, PCR cannot differentiate between living and dead bacteria. Furthermore, RT-PCR can be used to confirm the viability of the microorganisms [73]. Another study, by Saleh Mghir et al. [97], sought the development of mutant strains in their positive cultures, defined as having a three-fold-increased MIC. Infection was further analyzed by Wang et al. [78] by measuring a type II transmembrane protein FAP. FAP is involved in infection response and inflammation and is expressed when cells are under pressure [7]. Mao et al. [122] used the total weight of the infected synovial tissue (pus) as a measurement of infection. Fluorochrome staining was used to evaluate bone growth over time without sacrificing the rabbits. Several calcium-binding fluorophores can be injected at different time points. Four studies included in this review used two time points [102,104] or three time points [115,127]. Fluorescence microscopy is used after sacrificing the rabbits for visualizing dynamic bone growth. Mechanical testing of the excised limb was performed to evaluate the bone–implant integration and stability of the implant in several studies included in this review. Wang et al. [78] and Zhou et al. [116] tested the pull-out strength of the implant, and Schroeder et al. [98] measured the displacement of the implant after loading. Additional outcome measures may provide relevant information and aid the translation of PJI interventions to the clinic. Especially in longer studies, more information about bone ingrowth of the implant provides relevant information on how the implant might function in the clinic.

### 4.4. Limitations

Systematic reviews are valuable tools to evaluate and summarize multiple articles; however, they are not without their limitations. Every systematic review has the limitation of publication bias. Positive results are more likely to be published compared to studies without statistical significance, skewing the overall findings [162]. Positive studies may even result in multiple publications, as also seen in this review by multiple publications by the same author. As in most systematic reviews, language bias was also apparent in this review, as only English articles were included. Furthermore, comparing articles is difficult, as multiple articles have omitted information. This is shown by the number of cells that are empty in Table 1, Table 2, Table 3, Table 4 and Table 5 and the percentages of ‘not stated’ in Figure 3, Figure 4 and Figure 5. If articles had followed the ARRIVE guidelines, comparisons would have been more straightforward [43,44]. The overall quality of a systematic review is influenced by the quality of the studies included. Despite incomplete reporting, an overview of the available evidence is still valuable, though combining results and drawing strong conclusions may be challenging. Among the articles that information could be extracted from, heterogeneity was high. The methodology of a study was often adapted to the tested antibacterial technology. Differences in study populations, interventions, outcomes, and methodologies can make it difficult to combine and interpret results. These limitations show the importance of following guidelines set for in vivo experiments.

### 4.5. Animal-Free Science

Implementation of Russel and Burch’s 3R principles of reduction, refinement, and replacement should be used in all studies [38]. Optimization of bias control, the experimental design, and choosing the right outcome measures result in reduction and refinement. However, replacement is not discussed yet. Good in vitro data for antimicrobial activity and biocompatibility are needed to move on to in vivo or clinical experiments. Several reviews give an overview of the current availability of in vitro experiments to test the antimicrobial properties of new technologies. Methods range from simple static disc diffusion tests to more complex systems that take flow displacement into account [22,163,164]. All reviews conclude there is no one golden standard to use, especially since the working mechanisms of all antimicrobial compounds differ. As stated previously, several important in vivo factors cannot be replicated or integrated easily in vitro, which makes the translation from in vitro to in vivo challenging [23]. The lab-on-a-chip (LOC) technique has become more popular in the medical sector, aiming to bridge the gap between in vitro and in vivo systems. To simulate bone remodeling, cells are combined with mechanical, electrophysical, and biological stimuli, recreating cellular-, tissue-, and organ-level processes [165,166]. Currently, LOC has been used to simulate bone remodeling; however, to simulate PJI, bacteria would also have to be considered in the system. Although LOC is a promising technique for the future, currently, in vivo experiments are still needed before clinical studies. All antimicrobial compounds should be tested thoroughly in vitro to minimize the use of animals as much as possible. Unfortunately, complete replacement of animals is not possible yet.

### 4.6. Checklist for the Assessment of PJI in an In Vivo NZW Rabbit Model

To improve reproducibility and be able to compare studies to each other better, compliance with certain standards is necessary. During the 2023 international consensus meeting on musculoskeletal infection (MSKI), the need for unified and standardized criteria for animal testing in the treatment of MSKI was expressed [41].

Several guidelines already exist, like the ARRIVE guidelines [44], a checklist for publishing in vivo studies for orthopedic device-related infections by Moriarty et al. [24], and the Gold Standard Publication Checklist [64]. These lists have been adapted in Table 8, including the main points of this systematic review. Adhering to this checklist will potentially result in reproducible studies with limited bias, improving the overall quality of research. Furthermore, this checklist takes the 3Rs into account, resulting in less animal suffering.

**Table 8:** Checklist for setting up an in vivo NZW rabbit model for the assessment of PJI.

Aspect	Includes
**Bias control**	
Bias control	Blinding
	Randomization
	Humane endpoints (scoring sheets should be used to check if humane endpoints are met)
Rabbit characteristics	Sex (justification if not mixed male/female)
	Age (rabbits should be skeletally mature)
	Weight (rabbits should be skeletally mature)
Caretaking	Eating and supplemental feed
	Drinking
	Housing conditions
**Experimental design**	
General	Aim of study
	Antimicrobial technology tested
	Duration of study (based on study aim and working mechanism intervention)
	Total number of rabbits used
	Experimental groups and size (based on power calculations. Control group must always be included)
	Dropout number
	Acclimatization period
	Use of prophylactic antibiotics
Inoculum	Strain (explain why this species and strain)
	Inoculum size (explain size, report both CFU/mL and total volume used)
	Administration method (should mimic clinical situation)
	Containment method used or not
Implant	Description (size)
	Material
	Movement prevention
	Location
**Outcomes**	
Bacterial culture	Tissue cultured (specify what and how much tissue was used)
	Culture method
	Outcome unit
Health monitoring	Weight
	Temperature
	Clinical signs of infection
Hematology	CRP
	ESR
	WBC/leukocyte differentiation
	IL-6
Histology	Staining and tissues colored
Imaging	Method and outcome parameters
	Pre- or post-mortem
Other	Specify what/how outcome is measured, and what the link is to the study aim

## 5. Conclusions

In vivo NZW rabbit models can aid in studying new antibacterial technologies and PJI prevention. However, consensus in bias control, experimental design, outcome measures, and documentation thereof is missing. Regarding bias control and complying with the 3Rs, standardized guidelines are necessary. Blinding and randomization are essential to minimize bias and should consistently be implemented in the rabbit models. Furthermore, documentation of rabbit characteristics and animal caretaking is necessary to ensure scientific integrity, reliability, and reproducibility. The exact methodology and outcome parameters to be studied depend on the working mechanism and intended use of the antibacterial technique. Therefore, there is no gold standard in setting up these experiments. *S. aureus* might be the most logical option to inoculate due to its high prevalence in PJI; however, researchers should consider their research aim when choosing the pathogen, especially with regard to clinical or resistant strains. Determining the experimental design is crucial to better bridge the gap from in vivo experiments to the clinic. Given the wide range of potential outcome measures, the scientific value gained from including additional outcome measures must justify the increased burden on the animals or a larger group size. At a minimum, studies investigating new antibacterial technologies against PJI in a rabbit model should include bacterial culture, including documentation of the tissue cultured, the culture method, the outcome unit, and health monitoring. This review provides an overview of experimental requirements and outlines what should be documented and published for all NZW rabbit PJI models, based on and modified from existing guidelines, like the ARRIVE guidelines. Ultimately, this analysis aims to assist researchers in determining suitable clinically relevant methodologies and outcome measures for in vivo PJI models using NZW rabbits to test new antimicrobial technologies.

## Data Availability

The original contributions presented in this study are included in the article; further inquiries can be directed to the corresponding author.

## Appendix A

### Appendix A.1. Scopus Search String

TITLE-ABS-KEY({prosthesis-related infections } OR {PJI} OR {prosthetic joint infection} OR {joint replacement infection} OR {arthroplasty infection} OR {implant infection} OR ({osteomyelitis} AND {implant}) OR ({orthopaedic infection} AND {implant}) OR ({bone infection} AND {implant}) OR {prosthetic infection} OR {peri-prosthetic infection} OR {implant-related infection} OR {DAIR}) AND TITLE-ABS-KEY (rabbit* OR {lagomorpha} OR {new zealand white} OR rodent*) AND TITLE-ABS-KEY ({experimental model} OR {animal model} OR {preclinical model} OR {in vivo}) AND (LIMIT-TO (DOCTYPE,”ar”)) AND (LIMIT-TO (LANGUAGE,”English”)).

### Appendix A.2. EMBASE Search String

**Table A1:** EMBASE database search string.

Number	Search Term
N1	“prosthesis-related infections” OR “PJI” OR “prosthetic joint infection” OR “joint replacement infection” OR “arthroplasty infection” OR “implant infection” OR (“osteomyelitis” AND “implant”) OR (“orthopaedic infection” AND “implant”) OR (“bone infection” AND “implant”) OR “prosthetic infection” OR “peri-prosthetic infection” OR “implant-related infection” OR “DAIR”
N2	“rabbit*” OR “lagomorpha” OR “new zealand white” OR “rodent*”
N3	“model” OR “experimental model” OR “animal model” OR “preclinical model” OR “in vivo”
N4	N1 AND N2 AND N3

## References

[1] Parvizi J., Fassihi S.C., Enayatollahi M.A. (2016). Diagnosis of Periprosthetic Joint Infection Following Hip and Knee Arthroplasty. Orthop. Clin. N. Am..

[2] Sabah S.A., Alvand A., Price A.J. (2021). Revision knee replacement for prosthetic joint infection: Epidemiology, clinical outcomes and health-economic considerations. Knee.

[3] Klug A., Gramlich Y., Rudert M., Drees P., Hoffmann R., Weissenberger M., Kutzner K.P. (2021). The projected volume of primary and revision total knee arthroplasty will place an immense burden on future health care systems over the next 30 years. Knee Surg. Sports Traumatol. Arthrosc..

[4] Kurtz S., Ong K., Lau E., Mowat F., Halpern M. (2007). Projections of primary and revision hip and knee arthroplasty in the United States from 2005 to 2030. J. Bone Jt. Surg..

[5] Corvec S., Portillo M.E., Pasticci B.M., Borens O., Trampuz A. (2012). Epidemiology and new developments in the diagnosis of prosthetic joint infection. Int. J. Artif. Organs.

[6] Del Pozo J.L., Patel R. (2009). Infection associated with prosthetic joints. N. Engl. J. Med..

[7] Davidson D.J., Spratt D., Liddle A.D. (2019). Implant materials and prosthetic joint infection: The battle with the biofilm. EFORT Open Rev..

[8] Aggarwal V.K., Rasouli M.R., Parvizi J. (2013). Periprosthetic joint infection: Current concept. Indian J. Orthop..

[9] Wei H., Song X., Liu P., Liu X., Yan X., Yu L. (2022). Antimicrobial coating strategy to prevent orthopaedic device-related infections: Recent advances and future perspectives. Biomater. Adv..

[10] Xu Y., Huang T.B., Schuetz M.A., Choong P.F. (2023). Mortality, patient-reported outcome measures, and the health economic burden of prosthetic joint infection. EFORT Open Rev..

[11] Arciola C.R., An Y., Campoccia D., Donati M., Montanaro L. (2005). Etiology of implant orthopedic infections: A survey on 1027 clinical isolates. Int. J. Artif. Organs.

[12] Da Silva R.B., Salles M.J. (2021). Outcomes and risk factors in prosthetic joint infections by multidrug-resistant gram-negative bacteria: A retrospective cohort study. Antibiotics.

[13] Khatoon Z., McTiernan C.D., Suuronen E.J., Mah T.-F., Alarcon E.I. (2018). Bacterial biofilm formation on implantable devices and approaches to its treatment and prevention. Heliyon.

[14] Lazic I., Scheele C., Pohlig F., von Eisenhart-Rothe R., Suren C. (2021). Treatment options in PJI–is two-stage still gold standard?. J. Orthop..

[15] Arciola C.R., Campoccia D., Montanaro L. (2018). Implant infections: Adhesion, biofilm formation and immune evasion. Nat. Rev. Microbiol..

[16] Subbiahdoss G., Kuijer R., Grijpma D.W., van der Mei H.C., Busscher H.J. (2009). Microbial biofilm growth vs. tissue integration: “the race for the surface” experimentally studied. Acta Biomater..

[17] Bhattacharya M., Wozniak D.J., Stoodley P., Hall-Stoodley L. (2015). Prevention and treatment of *Staphylococcus aureus* biofilms. Expert Rev. Anti-Infect. Ther..

[18] Paharik A.E., Horswill A.R. (2016). The staphylococcal biofilm: Adhesins, regulation, and host response. Virulence Mech. Bact. Pathog..

[19] Yang S., Li X., Cang W., Mu D., Ji S., An Y., Wu R., Wu J. (2023). Biofilm tolerance, resistance and infections increasing threat of public health. Microb. Cell.

[20] Salam M.A., Al-Amin M.Y., Salam M.T., Pawar J.S., Akhter N., Rabaan A.A., Alqumber M.A.A. (2023). Antimicrobial Resistance: A Growing Serious Threat for Global Public Health. Healthcare.

[21] Amann S., Neef K., Kohl S. (2019). Antimicrobial resistance (AMR). Eur. J. Hosp. Pharm..

[22] Sjollema J., Zaat S.A.J., Fontaine V., Ramstedt M., Luginbuehl R., Thevissen K., Li J., van der Mei H.C., Busscher H.J. (2018). In vitro methods for the evaluation of antimicrobial surface designs. Acta Biomater..

[23] Moriarty T.F., Grainger D.W., Richards R.G. (2014). Challenges in linking preclinical anti-microbial research strategies with clinical outcomes for device-associated infections. Eur. Cell Mater..

[24] Moriarty T.F., Harris L.G., Mooney R.A., Wenke J.C., Riool M., Zaat S.A.J., Moter A., Schaer T.P., Khanna N., Kuehl R. (2019). Recommendations for design and conduct of preclinical in vivo studies of orthopedic device-related infection. J. Orthop. Res..

[25] Bevers R.T., van de Voort M.H., van Loo I.H., Geurts J., Arts J.J. (2022). The role of material technologies targeting *P. aeruginosa* and *S. aureus* quorum sensing in biofilm formation. Med. Res. Arch..

[26] Berti A., Rose W., Nizet V., Sakoulas G. (2020). Antibiotics and innate immunity: A cooperative effort toward the successful treatment of infections. Open Forum Infectious Diseases.

[27] Beer J., Wagner C.C., Zeitlinger M. (2009). Protein binding of antimicrobials: Methods for quantification and for investigation of its impact on bacterial killing. AAPS J..

[28] Sakai R., Takahashi A., Takahira N., Uchiyama K., Yamamoto T., Uchida K., Fukushima K., Moriya M., Takaso M., Itoman M. (2011). Hammering force during cementless total hip arthroplasty and risk of microfracture. Hip Int..

[29] Odekerken J.C.E. (2013). Modern Orthopaedic Implant Coatings—Their Pro’s, Con’s and Evaluation Methods. Mod. Surf. Eng. Treat..

[30] Yagi H., Kihara S., Mittwede P.N., Maher P.L., Rothenberg A.C., Falcione A.D.C.M., Chen A., Urish K.L., Tuan R.S., Alexander P.G. (2021). Development of a large animal rabbit model for chronic periprosthetic joint infection. Bone Jt. Res..

[31] Pearce A., Richards R., Milz S., Schneider E., Pearce S. (2007). Animal models for implant biomaterial research in bone: A review. Eur. Cell Mater..

[32] Hickman D., Johnson J., Vemulapalli T.H., Crisler J., Shepherd R. (2017). Commonly used animal models. Princ. Anim. Res. Grad. Undergrad. Stud..

[33] Mapara M., Thomas B.S., Bhat K. (2012). Rabbit as an animal model for experimental research. Dent. Res. J..

[34] Gatin L., Saleh-Mghir A., Massin P., Crémieux A.-C. (2015). Critical analysis of experimental models of periprosthetic joint infection. Orthop. Traumatol. Surg. Res..

[35] Bottagisio M., Coman C., Lovati A.B. (2019). Animal models of orthopaedic infections. A review of rabbit models used to induce long bone bacterial infections. J. Med. Microbiol..

[36] ARRIVE Guidelines. https://arriveguidelines.org/.

[37] Voehringer P., Nicholson J.R. (2019). Minimum information in In Vivo research. Handb. Exp. Pharmacol..

[38] Tannenbaum J., Bennett B.T. (2015). Russell and Burch’s 3Rs then and now: The need for clarity in definition and purpose. J. Am. Assoc. Lab. Anim. Sci..

[39] Page M.J., McKenzie J.E., Bossuyt P.M., Boutron I., Hoffmann T.C., Mulrow C.D., Shamseer L., Tetzlaff J.M., Akl E.A., Brennan S.E. (2021). The PRISMA 2020 statement: An updated guideline for reporting systematic reviews. BMJ.

[40] Covidence Systematic Review Software. www.covidence.org.

[41] Jennings J.A., Arts J.J., Abuhussein E., Alt V., Ashton N., Baertl S., Bhattacharyya S., Cain J.D., Dintakurthi Y., Ducheyne P. (2024). 2023 International Consensus Meeting on musculoskeletal infection: Summary from the treatment workgroup and consensus on treatment in preclinical models. J. Orthop. Res..

[42] Laajala T.D., Jumppanen M., Huhtaniemi R., Fey V., Kaur A., Knuuttila M., Aho E., Oksala R., Westermarck J., Mäkelä S. (2016). Optimized design and analysis of preclinical intervention studies in vivo. Sci. Rep..

[43] Kilkenny C., Browne W., Cuthill I.C., Emerson M., Altman D.G. (2011). Animal Research: Reporting In Vivo Experiments—The ARRIVE Guidelines.

[44] Kilkenny C., Browne W.J., Cuthill I.C., Emerson M., Altman D.G. (2014). Improving bioscience research reporting: The arrive guidelines for reporting animal research. Animals.

[45] Auer J.A., Goodship A., Arnoczky S., Pearce S., Price J., Claes L., Von Rechenberg B., Hofmann-Amtenbrinck M., Schneider E., Müller-Terpitz R. (2007). Refining animal models in fracture research: Seeking consensus in optimising both animal welfare and scientific validity for appropriate biomedical use. BMC Musculoskelet. Disord..

[46] Bespalov A., Wicke K., Castagné V. (2019). Blinding and randomization. Good Research Practice in Non-Clinical Pharmacology and Biomedicine.

[47] Fenwick N., Griffin G., Gauthier C. (2009). The welfare of animals used in science: How the “Three Rs” ethic guides improvements. Can. Vet. J..

[48] Huang W., Percie du Sert N., Vollert J., Rice A.S. (2020). General principles of preclinical study design. Good Research Practice in Non-Clinical Pharmacology and Biomedicine.

[49] Kilkenny C., Parsons N., Kadyszewski E., Festing M.F., Cuthill I.C., Fry D., Hutton J., Altman D.G. (2009). Survey of the quality of experimental design, statistical analysis and reporting of research using animals. PLoS ONE.

[50] Miller L.R., Marks C., Becker J.B., Hurn P.D., Chen W.J., Woodruff T., McCarthy M.M., Sohrabji F., Schiebinger L., Wetherington C.L. (2017). Considering sex as a biological variable in preclinical research. FASEB J..

[51] Kunutsor S.K., Whitehouse M.R., Blom A.W., Beswick A.D., Team I. (2016). Patient-related risk factors for periprosthetic joint infection after total joint arthroplasty: A systematic review and meta-analysis. PLoS ONE.

[52] Sukoff Rizzo S.J., McTighe S., McKinzie D.L. (2020). Genetic background and sex: Impact on generalizability of research findings in pharmacology studies. Good Research Practice in Non-Clinical Pharmacology and Biomedicine.

[53] Tannenbaum C., Ellis R.P., Eyssel F., Zou J., Schiebinger L. (2019). Sex and gender analysis improves science and engineering. Nature.

[54] Clayton J.A. (2016). Studying both sexes: A guiding principle for biomedicine. FASEB J..

[55] Thurston S., Burlingame L., Lester P.A., Lofgren J. (2018). Methods of pairing and pair maintenance of New Zealand White rabbits (*Oryctolagus cuniculus*) via behavioral ethogram, monitoring, and interventions. JoVE (J. Vis. Exp.).

[56] Mironenko C.M., Kapadia M., Donlin L., Figgie M., Carli A.V., Henry M., Goodman S.M., Miller A.O. (2021). 239. Sex Differences in Prosthetic Joint Infection. Open Forum Infectious Diseases.

[57] Higuera-Rueda C.A., Piuzzi N.S., Milbrandt N.B., Tsai Y.H., Klika A.K., Samia A.C.S., Visperas A. (2024). The Mark Coventry Award: PhotothermAA Gel Combined with Debridement, Antibiotics, and Implant Retention (DAIR) Significantly Decreases Implant Biofilm Burden and Soft-Tissue Infection in a Rabbit Model of Knee Periprosthetic Joint Infection. J. Arthroplast..

[58] Visperas A., Santana D., Ju M., Milbrandt N.B., Tsai Y.H., Wickramasinghe S., Klika A.K., Piuzzi N.S., Samia A.C.S., Higuera-Rueda C.A. (2022). Standardized quantification of biofilm in a novel rabbit model of periprosthetic joint infection. J. Bone Jt. Infect..

[59] Weisbroth S.H., Flatt R.E., Kraus A.L. (2013). The Biology of the Laboratory Rabbit.

[60] Marchandeau S., Pontier D., Guitton J.-S., Letty J., Fouchet D., Aubineau J., Berger F., Léonard Y., Roobrouck A., Gelfi J. (2014). Early infections by myxoma virus of young rabbits (*Oryctolagus cuniculus*) protected by maternal antibodies activate their immune system and enhance herd immunity in wild populations. Vet. Res..

[61] Attili A.-R., Nebbia P., Bellato A., Galosi L., Papeschi C., Rossi G., Linardi M., Fileni E., Cuteri V., Chiesa F. (2020). The effect of age and sampling site on the outcome of *Staphylococcus aureus* infection in a rabbit (*Oryctolagus cuniculus*) farm in Italy. Animals.

[62] Masoud I., Shapiro F., Kent R., Moses A. (1986). A longitudinal study of the growth of the New Zealand white rabbit: Cumulative and biweekly incremental growth rates for body length, body weight, femoral length, and tibial length. J. Orthop. Res..

[63] Dutta S., Sengupta P. (2018). Rabbits and men: Relating their ages. J. Basic Clin. Physiol. Pharmacol..

[64] Hooijmans C.R., Leenaars M., Ritskes-Hoitinga M. (2010). A gold standard publication checklist to improve the quality of animal studies, to fully integrate the Three Rs, and to make systematic reviews more feasible. Altern. Lab. Anim..

[65] Hooijmans C.R., de Vries R., Leenaars M., Curfs J., Ritskes-Hoitinga M. (2011). Improving planning, design, reporting and scientific quality of animal experiments by using the Gold Standard Publication Checklist, in addition to the ARRIVE guidelines. Br. J. Pharmacol..

[66] European Parliament, European Council (2010). 63/EU of the European Parliament and of the Council of 22 September 2010 on the protection of animals used for scientific purposes. Off. J. Eur. Union.

[67] Stokes W.S. (2002). Humane endpoints for laboratory animals used in regulatory testing. ILAR J..

[68] Department of Animals in Science and Society, Faculty of Veterinary Medicine Utrecht University Humane Endpoints in Laboratory Animal Experimentation. https://www.humane-endpoints.info/en.

[69] Craig M.R., Poelstra K.A., Sherrell J.C., Kwon M.S., Belzile E.L., Brown T.E. (2005). A novel total knee arthroplasty infection model in rabbits. J. Orthop. Res..

[70] Gahukamble A.D., McDowell A., Post V., Salavarrieta Varela J., Rochford E.T., Richards R.G., Patrick S., Moriarty T.F. (2014). *Propionibacterium acnes* and *Staphylococcus lugdunensis* cause pyogenic osteomyelitis in an intramedullary nail model in rabbits. J. Clin. Microbiol..

[71] López T., Sanz-Ruíz P., Navarro-García F., León-Román V.E., Vaquero-Martín J. (2020). Experimental reproduction of periprosthetic joint infection: Developing a representative animal model. Knee.

[72] Odekerken J.C., Brans B.T., Welting T.J., Walenkamp G.H. (2014). (18)F-FDG microPET imaging differentiates between septic and aseptic wound healing after orthopedic implant placement: A longitudinal study of an implant osteomyelitis in the rabbit tibia. Acta Orthop..

[73] Poultsides L.A., Papatheodorou L.K., Karachalios T.S., Khaldi L., Maniatis A., Petinaki E., Malizos K.N. (2008). Novel model for studying hematogenous infection in an experimental setting of implant-related infection by a community-acquired methicillin-resistant *S. aureus* strain. J. Orthop. Res..

[74] Sarda L., Saleh-Mghir A., Peker C., Meulemans A., Cremieux A.C., Le Guludec D. (2002). Evaluation of 99mTc-ciprofloxacin scintigraphy in a rabbit model of *Staphylococcus aureus* prosthetic joint infection. J. Nucl. Med..

[75] Sarda-Mantel L., Saleh-Mghir A., Welling M.M., Meulemans A., Vrigneaud J.M., Raguin O., Hervatin F., Martet G., Chau F., Lebtahi R. (2007). Evaluation of 99mTc-UBI 29-41 scintigraphy for specific detection of experimental *Staphylococcus aureus* prosthetic joint infections. Eur. J. Nucl. Med. Mol. Imaging.

[76] Tang C., Wang F., Hou Y., Lu S., Tian W., Xu Y., Jin C., Wang L. (2015). Technetium-99m-labeled annexin V imaging for detecting prosthetic joint infection in a rabbit model. J. Biomed. Res..

[77] Wang Y., Liu H., Yao S., Guan Z., Li Q., Qi E., Li X., Zhang J., Tian J. (2022). Using18F-flurodeoxyglucose and68Ga-fibroblast activation protein inhibitor PET/CT to evaluate a new periprosthetic joint infection model of rabbit due to *Staphylococcus aureus*. Nucl. Med. Commun..

[78] Wang Y., Li Y., Han L., Wang J., Zhang C., Qi E., Zhang D., Zhang X., Huan Y., Tian J. (2022). 18F-FDG and 68 Ga-FAPI PET/CT for the evaluation of periprosthetic joint infection and aseptic loosening in rabbit models. BMC Musculoskelet. Disord..

[79] Brunotte M., Rupp M., Stötzel S., Sommer U., Mohammed W., Thormann U., Heiss C., Lips K.S., Domann E., Alt V. (2019). A new small animal model for simulating a two-stage-revision procedure in implant-related methicillin-resistant *Staphylococcus aureus* bone infection. Injury.

[80] Efstathopoulos N., Giamarellos-Bourboulis E., Kanellakopoulou K., Lazarettos I., Giannoudis P., Frangia K., Magnissalis E., Papadaki M., Nikolaou V.S. (2008). Treatment of experimental osteomyelitis by Methicillin Resistant *Staphylococcus aureus* with bone cement system releasing grepafloxacin. Injury.

[81] Gatin L., Mghir A.S., Mouton W., Laurent F., Ghout I., Rioux-Leclercq N., Tattevin P., Verdier M.C., Cremieux A.C. (2019). Colistin-containing cement spacer for treatment of experimental carbapenemase-producing *Klebsiella pneumoniae* prosthetic joint infection. Int. J. Antimicrob. Agents.

[82] Ismael F., Bléton R., Saleh-Mghir A., Dautrey S., Massias L., Crémieux A.C. (2003). Teicoplanin-containing cement spacers for treatment of experimental *Staphylococcus aureus* joint prosthesis infection. Antimicrob. Agents Chemother..

[83] López T., Vaquero-Martín J., Torres-Suárez A.I., Navarro-García F., Fraguas-Sánchez A.I., León-Román V.E., Sanz-Ruíz P. (2022). The tale of microencapsulated rifampicin: Is it useful for the treatment of periprosthetic joint infection?. Int. Orthop..

[84] Nijhof M.W., Fleer A., Hardus K., Vogely H.C., Schouls L.M., Verbout A.J., Dhert W.J. (2001). Tobramycin-containing bone cement and systemic cefazolin in a one-stage revision. Treatment of infection in a rabbit model. J. Biomed. Mater. Res..

[85] Overstreet D., McLaren A., Calara F., Vernon B., McLemore R. (2015). Local gentamicin delivery from resorbable viscous hydrogels is therapeutically effective. Clin. Orthop. Relat. Res..

[86] Yan S., Cai X., Yan W., Dai X., Wu H. (2007). Continuous wave ultrasound enhances vancomycin release and antimicrobial efficacy of antibiotic-loaded acrylic bone cement in vitro and in vivo. J. Biomed. Mater. Res. B Appl. Biomater..

[87] Ambrose C.G., Clyburn T.A., Mika J., Gogola G.R., Kaplan H.B., Wanger A., Mikos A.G. (2014). Evaluation of antibiotic-impregnated microspheres for the prevention of implant-associated orthopaedic infections. J. Bone Jt. Surg. Am..

[88] Aykut S., Oztürk A., Ozkan Y., Yanik K., Ilman A.A., Ozdemir R.M. (2010). Evaluation and comparison of the antimicrobial efficacy of teicoplanin- and clindamycin-coated titanium implants: An experimental study. J. Bone Jt. Surg. Br..

[89] Darouiche R.O., Mansouri M.D., Zakarevicz D., Alsharif A., Landon G.C. (2007). In vivo efficacy of antimicrobial-coated devices. J. Bone Jt. Surg. Am..

[90] Gatin L., Saleh-Mghir A., Tasse J., Ghout I., Laurent F., Crémieux A.C. (2014). Ceftaroline-Fosamil efficacy against methicillin-resistant *Staphylococcus aureus* in a rabbit prosthetic joint infection model. Antimicrob. Agents Chemother..

[91] Giavaresi G., Meani E., Sartori M., Ferrari A., Bellini D., Sacchetta A.C., Meraner J., Sambri A., Vocale C., Sambri V. (2014). Efficacy of antibacterial-loaded coating in an in vivo model of acutely highly contaminated implant. Int. Orthop..

[92] Helbig L., Simank H.G., Lorenz H., Putz C., Wölfl C., Suda A.J., Moghaddam A., Schmidmaier G., Guehring T. (2014). Establishment of a new methicillin resistant *Staphylococcus aureus* animal model of osteomyelitis. Int. Orthop..

[93] Li D., Lv P., Fan L., Huang Y., Yang F., Mei X., Wu D. (2017). The immobilization of antibiotic-loaded polymeric coatings on osteoarticular Ti implants for the prevention of bone infections. Biomater. Sci..

[94] Liu D., He C., Liu Z., Xu W. (2017). Gentamicin coating of nanotubular anodized titanium implant reduces implant-related osteomyelitis and enhances bone biocompatibility in rabbits. Int. J. Nanomed..

[95] Muller-Serieys C., Saleh Mghir A., Massias L., Fantin B. (2009). Bactericidal activity of the combination of levofloxacin with rifampin in experimental prosthetic knee infection in rabbits due to methicillin-susceptible *Staphylococcus aureus*. Antimicrob. Agents Chemother..

[96] Saleh-Mghir A., Ameur N., Muller-Serieys C., Ismael F., Lemaitre F., Massias L., Feger C., Bléton R., Crémieux A.C. (2002). Combination of quinupristin-dalfopristin (synercid) and rifampin is highly synergistic in experimental *Staphylococcus aureus* joint prosthesis infection. Antimicrob. Agents Chemother..

[97] Saleh-Mghir A., Muller-Serieys C., Dinh A., Massias L., Crémieux A.C. (2011). Adjunctive rifampin is crucial to optimizing daptomycin efficacy against rabbit prosthetic joint infection due to methicillin-resistant *Staphylococcus aureus*. Antimicrob. Agents Chemother..

[98] Schroeder K., Simank H.G., Lorenz H., Swoboda S., Geiss H.K., Helbig L. (2012). Implant stability in the treatment of MRSA bone implant infections with linezolid versus vancomycin in a rabbit model. J. Orthop. Res..

[99] Auñón Á., Esteban J., Doadrio A.L., Boiza-Sánchez M., Mediero A., Eguibar-Blázquez D., Cordero-Ampuero J., Conde A., Arenas M., de-Damborenea J.J. (2020). *Staphylococcus aureus* Prosthetic Joint Infection Is Prevented by a Fluorine- and Phosphorus-Doped Nanostructured Ti-6Al-4V Alloy Loaded With Gentamicin and Vancomycin. J. Orthop. Res..

[100] Horn J., Schlegel U., Krettek C., Ito K. (2005). Infection resistance of unreamed solid, hollow slotted and cannulated intramedullary nails: An in-vivo experimental comparison. J. Orthop. Res..

[101] Moriarty T.F., Campoccia D., Nees S.K., Boure L.P., Richards R.G. (2010). In vivo evaluation of the effect of intramedullary nail microtopography on the development of local infection in rabbits. Int. J. Artif. Organs.

[102] Ravanetti F., Chiesa R., Ossiprandi M.C., Gazza F., Farina V., Martini F.M., Di Lecce R., Gnudi G., Della Valle C., Gavini J. (2016). Osteogenic response and osteoprotective effects in vivo of a nanostructured titanium surface with antibacterial properties. J. Mater. Sci. Mater. Med..

[103] Bitika O., Uzuna H., Kecika A. (2013). In-vivo analysis of antibacterial silver coated titanium implants in a contaminated rabbit knee model. Turk. Klin. J. Med. Sci..

[104] Boot W., Vogely H.C., Jiao C., Nikkels P.G., Pouran B., van Rijen M.H., Ekkelenkamp M.B., Hänsch G.M., Dhert W.J., Gawlitta D. (2020). Prophylaxis of implant-related infections by local release of vancomycin from a hydrogel in rabbits. Eur. Cell Mater..

[105] Fabritius M., Al-Munajjed A.A., Freytag C., Jülke H., Zehe M., Lemarchand T., Arts J.J., Schumann D., Alt V., Sternberg K. (2020). Antimicrobial silver multilayer coating for prevention of bacterial colonization of orthopedic implants. Materials.

[106] Kose N., Otuzbir A., Pekşen C., Kiremitçi A., Doğan A. (2013). A silver ion-doped calcium phosphate-based ceramic nanopowder-coated prosthesis increased infection resistance. Clin. Orthop. Relat. Res..

[107] Kose N., Çaylak R., Pekşen C., Kiremitçi A., Burukoglu D., Koparal S., Doğan A. (2016). Silver ion doped ceramic nano-powder coated nails prevent infection in open fractures: In vivo study. Injury.

[108] Metsemakers W.J., Emanuel N., Cohen O., Reichart M., Potapova I., Schmid T., Segal D., Riool M., Kwakman P.H.S., De Boer L. (2015). A doxycycline-loaded polymer-lipid encapsulation matrix coating for the prevention of implant-related osteomyelitis due to doxycycline-resistant methicillin-resistant *Staphylococcus aureus*. J. Control Release.

[109] Moojen D.J.F., Vogely H.C., Fleer A., Nikkels P.G.J., Higham P.A., Verbout A.J., Castelein R.M., Dhert W.J.A. (2009). Prophylaxis of infection and effects on osseointegration using a tobramycin-periapatite coating on titanium implants—An experimental study in the rabbit. J. Orthop. Res..

[110] Neut D., Dijkstra R.J.B., Thompson J.I., Kavanagh C., van der Mei H.C., Busscher H.J. (2015). A biodegradable gentamicin-hydroxyapatite-coating for infection prophylaxis in cementless hip prostheses. Eur. Cells Mater..

[111] Oosterbos C.J.M., Ch Vogely H., Nijhof M.W., Fleer A., Verbout A.J., Tonino A.J., Dhert W.J.A. (2002). Osseointegration of hydroxyapatite-coated and noncoated Ti6Al4V implants in the presence of local infection: A comparative histomorphometrical study in rabbits. J. Biomed. Mater. Res..

[112] Yang C.C., Lin C.C., Liao J.W., Yen S.K. (2013). Vancomycin-chitosan composite deposited on post porous hydroxyapatite coated Ti6Al4V implant for drug controlled release. Mater. Sci. Eng. C Mater. Biol. Appl..

[113] Zhang L., Yang Y., Zhang W., Lv H., Yang F., Lin C., Tang P. (2016). Inhibitory effect of super-hydrophobicity on silver release and antibacterial properties of super-hydrophobic Ag/TiO2 nanotubes. J. Biomed. Mater. Res.—Part B Appl. Biomater..

[114] Zhang C., Li X., Xiao D., Zhao Q., Chen S., Yang F., Liu J., Duan K. (2022). Cu^2+^ Release from Polylactic Acid Coating on Titanium Reduces Bone Implant-Related Infection. J. Funct. Biomater..

[115] Zhao F., Gao A., Liao Q., Li Y., Ullah I., Zhao Y., Ren X., Tong L., Li X., Zheng Y. (2024). Balancing the Anti-Bacterial and Pro-Osteogenic Properties of Ti-Based Implants by Partial Conversion of ZnO Nanorods into Hybrid Zinc Phosphate Nanostructures. Adv. Funct. Mater..

[116] Zhou J., Li B., Zhao L., Zhang L., Han Y. (2017). F-Doped Micropore/Nanorod Hierarchically Patterned Coatings for Improving Antibacterial and Osteogenic Activities of Bone Implants in Bacteria-Infected Cases. ACS Biomater. Sci. Eng..

[117] Zhou H., Ye S., Xu M., Hao L., Chen J., Fang Z., Guo K., Chen Y., Wang L. (2023). Dynamic surface adapts to multiple service stages by orchestrating responsive polymers and functional peptides. Biomaterials.

[118] Gilotra M., Nguyen T., Jaffe D., Sterling R. (2015). Dilute betadine lavage reduces implant-related bacterial burden in a rabbit knee prosthetic infection model. Am. J. Orthop..

[119] Heffernan J.M., Overstreet D.J., Vernon B.L., McLemore R.Y., Nagy T., Moore R.C., Badha V.S., Childers E.P., Nguyen M.B., Gentry D.D. (2022). In vivo evaluation of temperature-responsive antimicrobial-loaded PNIPAAm hydrogels for prevention of surgical site infection. J. Biomed. Mater. Res.—Part B Appl. Biomater..

[120] Komnos G., Banios K., Kolonia K., Poultsides L.A., Petinaki E., Sarrou S., Zintzaras E., Karachalios T. (2021). Do trabecular metal and cancellous titanium implants reduce the risk of late haematogenous infection? An experimental study in rabbits. HIP Int..

[121] Mäkinen T.J., Veiranto M., Knuuti J., Jalava J., Törmälä P., Aro H.T. (2005). Efficacy of bioabsorbable antibiotic containing bone screw in the prevention of biomaterial-related infection due to *Staphylococcus aureus*. Bone.

[122] Mao Y., Valour F., Nguyen N.T.Q., Doan T.M.N., Koelkebeck H., Richardson C., Cheng L.I., Sellman B.R., Tkaczyk C., Diep B.A. (2021). Multi-mechanistic Monoclonal Antibody Combination Targeting Key *Staphylococcus aureus* Virulence Determinants in a Rabbit Model of Prosthetic Joint Infection. Antimicrob. Agents Chemother..

[123] Yu B., Wen J.Q., Jiang Y., Chen Y., Yu G.N., Ren H.F., Ge D.Z., Wang Z.Y. (2019). Antibacterial activity of a novel titanium-copper (Ti-Cu) sintered alloy in preventing periprosthetic joint infection in rabbit model. J. Biomater. Tissue Eng..

[124] Zhai H., Pan J., Pang E., Bai B. (2014). Lavage with allicin in combination with vancomycin inhibits biofilm formation by *Staphylococcus epidermidis* in a rabbit model of prosthetic joint infection. PLoS ONE.

[125] Zhou B., Zhang D. (2018). Antibacterial effects of bacteriocins isolated from lactobacillus rhamnosus (ATCC 53103) in a rabbit model of knee implant infection. Exp. Ther. Med..

[126] Zhu Y., Weng X., Zhang J., Mao J. (2023). Protective effect of additional cathelicidin antimicrobial peptide PR-39 on prosthetic-joint infections. J. Orthop. Surg..

[127] Odekerken J.C., Arts J.J., Surtel D.A., Walenkamp G.H., Welting T.J. (2013). A rabbit osteomyelitis model for the longitudinal assessment of early post-operative implant infections. J. Orthop. Surg. Res..

[128] Hayakawa T., Yoshinari M., Kiba H., Yamamoto H., Nemoto K., Jansen J.A. (2002). Trabecular bone response to surface roughened and calcium phosphate (Ca-P) coated titanium implants. Biomaterials.

[129] Sul Y.T., Byon E.S.E.S., Jeong Y.Y. (2004). Biomechanical measurements of calcium-incorporated oxidized implants in rabbit bone: Effect of calcium surface chemistry of a novel implant. Clin. Implant Dent. Relat. Res..

[130] Breding K., Jimbo R., Hayashi M., Xue Y., Mustafa K., Andersson M. (2014). The effect of hydroxyapatite nanocrystals on osseointegration of titanium implants: An in vivo rabbit study. Int. J. Dent..

[131] Roberts W.E., Smith R.K., Zilberman Y., Mozsary P.G., Smith R.S. (1984). Osseous adaptation to continuous loading of rigid endosseous implants. Am. J. Orthod..

[132] Slaets E., Carmeliet G., Naert I., Duyck J. (2006). Early cellular responses in cortical bone healing around unloaded titanium implants: An animal study. J. Periodontol..

[133] Hermida J.C., Bergula A., Dimaano F., Hawkins M., Colwell C.W., D’Lima D.D. (2010). An in vivo evaluation of bone response to three implant surfaces using a rabbit intramedullary rod model. J. Orthop. Surg. Res..

[134] Peng H.-M., Zhou Z.-K., Wang F., Yan S.-G., Xu P., Shang X.-F., Zheng J., Zhu Q.-S., Cao L., Weng X.-S. (2021). Microbiology of periprosthetic hip and knee infections in surgically revised cases from 34 centers in mainland China. Infect. Drug Resist..

[135] Levy P.Y., Fenollar F., Stein A., Borrione F., Cohen E., Lebail B., Raoult D. (2008). *Propionibacterium acnes* postoperative shoulder arthritis: An emerging clinical entity. Clin. Infect. Dis..

[136] Sampathkumar P., Osmon D.R., Cockerill F.R. (2000). Prosthetic joint infection due to *Staphylococcus lugdunensis*. Mayo Clin. Proc..

[137] Shah N.B., Osmon D.R., Fadel H., Patel R., Kohner P.C., Steckelberg J.M., Mabry T., Berbari E.F. (2010). Laboratory and clinical characteristics of *Staphylococcus lugdunensis* prosthetic joint infections. J. Clin. Microbiol..

[138] Tsai Y., Chang C.-H., Lin Y.-C., Lee S.-H., Hsieh P.-H., Chang Y. (2019). Different microbiological profiles between hip and knee prosthetic joint infections. J. Orthop. Surg..

[139] WHO Publishes List of Bacteria for Which New Antibiotics Are Urgently Needed. https://www.who.int/news/item/27-02-2017-who-publishes-list-of-bacteria-for-which-new-antibiotics-are-urgently-needed.

[140] Eiselt V.A., Bereswill S., Heimesaat M.M. (2024). Phage therapy in prosthetic joint infections caused by *Staphylococcus aureus*—A literature review. Eur. J. Microbiol. Immunol..

[141] Tuchscherr L., Pöllath C., Siegmund A., Deinhardt-Emmer S., Hoerr V., Svensson C.-M., Thilo Figge M., Monecke S., Löffler B. (2019). Clinical *S. aureus* isolates vary in their virulence to promote adaptation to the host. Toxins.

[142] Guo G., Wang J., You Y., Tan J., Shen H. (2017). Distribution characteristics of *Staphylococcus* spp. in different phases of periprosthetic joint infection: A review. Exp. Ther. Med..

[143] Hischebeth G., Randau T., Ploeger M., Friedrich M., Kaup E., Jacobs C., Molitor E., Hoerauf A., Gravius S., Wimmer M. (2019). *Staphylococcus aureus* versus *Staphylococcus epidermidis* in periprosthetic joint infection—Outcome analysis of methicillin-resistant versus methicillin-susceptible strains. Diagn. Microbiol. Infect. Dis..

[144] Lora-Tamayo J., Murillo O., Iribarren J.A., Soriano A., Sánchez-Somolinos M., Baraia-Etxaburu J.M., Rico A., Palomino J., Rodríguez-Pardo D., Horcajada J.P. (2013). A large multicenter study of methicillin–susceptible and methicillin–resistant *Staphylococcus aureus* prosthetic joint infections managed with implant retention. Clin. Infect. Dis..

[145] Dreikausen L., Blender B., Trifunovic-Koenig M., Salm F., Bushuven S., Gerber B., Henke M. (2023). Analysis of microbial contamination during use and reprocessing of surgical instruments and sterile packaging systems. PLoS ONE.

[146] Guarch-Pérez C., Riool M., de Boer L., Kloen P., Zaat S. (2023). Bacterial reservoir in deeper skin is a potential source for surgical site and biomaterial-associated infections. J. Hosp. Infect..

[147] Rakow A., Perka C., Trampuz A., Renz N. (2019). Origin and characteristics of haematogenous periprosthetic joint infection. Clin. Microbiol. Infect..

[148] Zeller V., Kerroumi Y., Meyssonnier V., Heym B., Metten M.-A., Desplaces N., Marmor S. (2018). Analysis of postoperative and hematogenous prosthetic joint-infection microbiological patterns in a large cohort. J. Infect..

[149] Wagner E.R., Farley K.X., Higgins I., Wilson J.M., Daly C.A., Gottschalk M.B. (2020). The incidence of shoulder arthroplasty: Rise and future projections compared with hip and knee arthroplasty. J. Shoulder Elb. Surg..

[150] Ko M.J., Lim C.-Y. (2021). General considerations for sample size estimation in animal study. Korean J. Anesthesiol..

[151] Ahmad S.S., Shaker A., Saffarini M., Chen A.F., Hirschmann M.T., Kohl S. (2016). Accuracy of diagnostic tests for prosthetic joint infection: A systematic review. Knee Surg. Sports Traumatol. Arthrosc..

[152] Bugnon P., Heimann M., Thallmair M. (2016). What the literature tells us about score sheet design. Lab. Anim..

[153] Benato L., Murrell J., Rooney N. (2022). Bristol Rabbit Pain Scale (BRPS): Clinical utility, validity and reliability. BMC Vet. Res..

[154] Miller A.L., Leach M.C. (2023). Pain recognition in rabbits. Vet. Clin. Exot. Anim. Pract..

[155] Melillo A. (2007). Rabbit clinical pathology. J. Exot. Pet Med..

[156] Vaishya R., Sardana R., Butta H., Mendiratta L. (2019). Laboratory diagnosis of prosthetic joint infections: Current concepts and present status. J. Clin. Orthop. Trauma.

[157] Berbari E., Mabry T., Tsaras G., Spangehl M., Erwin P.J., Murad M.H., Steckelberg J., Osmon D. (2010). Inflammatory blood laboratory levels as markers of prosthetic joint infection: A systematic review and meta-analysis. JBJS.

[158] Brewer N.R. (2006). Biology of the rabbit. J. Am. Assoc. Lab. Anim. Sci..

[159] Van den Kieboom J., Bosch P., Plate J., IJpma F., Kuehl R., McNally M., Metsemakers W., Govaert G. (2018). Diagnostic accuracy of serum inflammatory markers in late fracture-related infection: A systematic review and meta-analysis. Bone Jt. J..

[160] Ashraf M.A., Goyal A. (2023). Fludeoxyglucose (18F). StatPearls [Internet].

[161] Andriesse G., Elberts S., Vrolijk A., Verhulst C., Kluytmans J. (2011). Evaluation of a fourth-generation latex agglutination test for the identification of *Staphylococcus aureus*. Eur. J. Clin. Microbiol. Infect. Dis..

[162] Yuan Y., Hunt R.H. (2009). Systematic reviews: The good, the bad, and the ugly. Off. J. Am. Coll. Gastroenterol. ACG.

[163] Schumacher A., Vranken T., Malhotra A., Arts J., Habibovic P. (2018). In vitro antimicrobial susceptibility testing methods: Agar dilution to 3D tissue-engineered models. Eur. J. Clin. Microbiol. Infect. Dis..

[164] Coenye T., Nelis H.J. (2010). In vitro and in vivo model systems to study microbial biofilm formation. J. Microbiol. Methods.

[165] George E.L., Truesdell S.L., York S.L., Saunders M.M. (2018). Lab-on-a-chip platforms for quantification of multicellular interactions in bone remodeling. Exp. Cell Res..

[166] Kim S., Rajendran A.K., Amirthalingam S., Kim J.H., So K.-H., Hwang N.S. (2023). Recent technological advances in lab-on-a-chip for bone remodeling. Biosens. Bioelectron. X.

